# *Helicobacter pylori* from Peruvian Amerindians: Traces of Human Migrations in Strains from Remote Amazon, and Genome Sequence of an Amerind Strain

**DOI:** 10.1371/journal.pone.0015076

**Published:** 2010-11-29

**Authors:** Dangeruta Kersulyte, Awdhesh Kalia, Robert H. Gilman, Melissa Mendez, Phabiola Herrera, Lilia Cabrera, Billie Velapatiño, Jacqueline Balqui, Freddy Paredes Puente de la Vega, Carlos A. Rodriguez Ulloa, Jaime Cok, Catherine C. Hooper, Giedrius Dailide, Sravya Tamma, Douglas E. Berg

**Affiliations:** 1 Department of Molecular Microbiology, Washington University School of Medicine, St. Louis, Missouri, United States of America; 2 Department of Biology, University of Louisville, Louisville, Kentucky, United States of America; 3 Departemento de Microbiologia, Universidad Peruana Cayetano Heredia, Lima, Peru; 4 Asociacion Benefica PRISMA, Lima, Peru; 5 Department of International Health, The Johns Hopkins Bloomberg School of Public Health, Baltimore, Maryland, United States of America; 6 Centro de Salud, Kepashiato, Cusco, Peru; 7 Policlinico Peruano Japones, Lima, Peru; 8 Departments of Genetics and Medicine, Washington University School of Medicine, St. Louis, Missouri, United States of America; University of Hyderabad, India

## Abstract

**Background:**

The gastric pathogen *Helicobacter pylori* is extraordinary in its genetic diversity, the differences between strains from well-separated human populations, and the range of diseases that infection promotes.

**Principal Findings:**

Housekeeping gene sequences from *H. pylori* from residents of an Amerindian village in the Peruvian Amazon, Shimaa, were related to, but not intermingled with, those from Asia. This suggests descent of Shimaa strains from *H. pylori* that had infected the people who migrated from Asia into The Americas some 15,000+ years ago. In contrast, European type sequences predominated in strains from Amerindian Lima shantytown residents, but with some 12% Amerindian or East Asian-like admixture, which indicates displacement of ancestral purely Amerindian strains by those of hybrid or European ancestry. The genome of one Shimaa village strain, Shi470, was sequenced completely. Its SNP pattern was more Asian- than European-like genome-wide, indicating a purely Amerind ancestry. Among its unusual features were two *cagA* virulence genes, each distinct from those known from elsewhere; and a novel allele of gene *hp0519*, whose encoded protein is postulated to interact with host tissue. More generally, however, the Shi470 genome is similar in gene content and organization to those of strains from industrialized countries.

**Conclusions:**

Our data indicate that Shimaa village *H. pylori* descend from Asian strains brought to The Americas many millennia ago; and that Amerind strains are less fit than, and were substantially displaced by, hybrid or European strains in less isolated communities. Genome comparisons of *H. pylori* from Amerindian and other communities should help elucidate evolutionary forces that have shaped pathogen populations in The Americas and worldwide.

## Introduction

The history of European conquests in The Americas illustrates the potentially huge impact that contact between once-separate human populations can have on public health if one population has not experienced pathogens that are common in the other. More than 80% of indigenous Amerindians died in the decades after initial European contact from viral diseases such as smallpox, measles and influenza that probably had been endemic in Europe and Asia for millennia but absent from pre-Columbian Amerindian populations [1]–[4]. We hypothesize that encounters between invading Europeans and resident Amerindians also affected populations of other less lethal pathogens. This view is tested here with isolates of *Helicobacter pylori*, a genetically diverse bacterial pathogen that chronically infects the stomachs of billions of people worldwide. *H. pylori* infection is particularly common in developing countries, and its modes of transmission and carriage differ markedly from those of the viruses listed above [5], [6].

*H. pylori* is implicated in stomach and duodenal ulcers and gastric cancer, and also in iron deficiency anemia and increased susceptibility to other gastrointestinal pathogens, although most infections are asymptomatic [7]–[9]. In addition, it has been suggested that some *H. pylori* infections are beneficial, helping protect against illnesses such as esophageal reflux disease, cancer of the cardia and esophagus, and tuberculosis [9], [10], although this idea is controversial [11]. The broad range of *H. pylori* infection outcomes is likely to stem from genetic differences among strains, along with differences in genotypes, physiologies and environments of their human hosts.

Residents of developing countries tend to be infected repeatedly throughout their lives with new *H. pylori* strains, often transmitted from unrelated people and other households in the community. Much of this inter-household transmission is likely to stem from deficiencies in sanitary infrastructure that underlie the generally high infectious disease burden among the very poor worldwide. In contrast, new *H. pylori* infection has become much less common in industrialized societies, and when it occurs at all, usually involves transmission from adult to child within the same family [5], [6], [12]–[14].

Analyses of representative housekeeping gene sequences have shown that independent *H. pylori* isolates from most communities are readily distinguished from one another; and that different sets of genotypes predominate in strains from well separated human populations, such as those of Western Europe, Eastern Asia and Sub-Saharan Africa [15], [16]. Much of this diversity can be ascribed to high rates of mutation and inter-strain recombination [17], [18]. Also important are *H. pylori*'s preferentially local transmission [5], [6], [12], [13], and the isolation by distance of ancient human populations [19] and thereby of the *H. pylori* they carry. Localized transmission diminishes gene flow between separate populations and thereby fosters divergence by random genetic drift and adaptation to local conditions. The striking geographic differences among *H. pylori* genotypes had initially suggested that *H. pylori* DNA sequences be used to help elucidate human ancestries and ancient migrations [20]–[22], although the thousands of informative human DNA polymorphisms identified in recent years now provide the principal markers for such ancestry studies [23], [24].

Latin American *H. pylori* strains provide an intriguing and important exception to the usual correlation between human and *H. pylori* ancestries. Early studies had identified insertion/deletion motifs that distinguished European and Asian strains, and showed that most strains from residents of a Lima (Peru) shantytown contained the European, not the Asian, motif [25]. In confirmation, the sequences of representative housekeeping genes also indicated that shantytown strains were mostly European-like [26]. These findings were noteworthy because the shantytown residents are predominantly Amerindian, the descendants of ancient people who probably migrated into The Americas from Asia *via* a Bering Straits land bridge some 15,000 or more years ago [23]. One explanation for the unexpected predominance of European-type sequences in shantytown *H. pylori* assumed that pre-Columbian Amerindians were *H. pylori*-free [25]. An alternative model holds that *H. pylori* were widespread among all ancient peoples, but that Amerind strains were less fit than, and were displaced by those of Europeans [27]. Support for this second model came from occasional findings of Asian-like DNA sequences in some Latin American strains [26,27; results presented below], although there is a possibility that some Asian-like sequences derive from strains of more recent East Asian immigrants (large numbers came to Latin America starting in the mid-1800s) [28], [29].

With this background, we analyzed *H. pylori* from residents of the remote Peruvian Amazonian village of Shimaa. Here we show that gene sequences of Shimaa strains fall into a unique phylogenetic cluster, related to, but distinct from those from East Asia; and report the finished genome sequence of a representative Shimaa strain (Shi470). This is complemented by the recently released genome sequence of a strain from a Venezuelan Amerindian [30]. Analyses of *H. pylori* strains from remote and urban communities should help elucidate evolutionary forces that operated on pathogen populations in The Americas pre- and post-conquest, and more generally, reveal how encounters between long-separated human populations can affect microbial populations, genome evolution and human disease.

## Results

### Distinctiveness and genetic diversity of Shimaa strains

**Figure 1:**
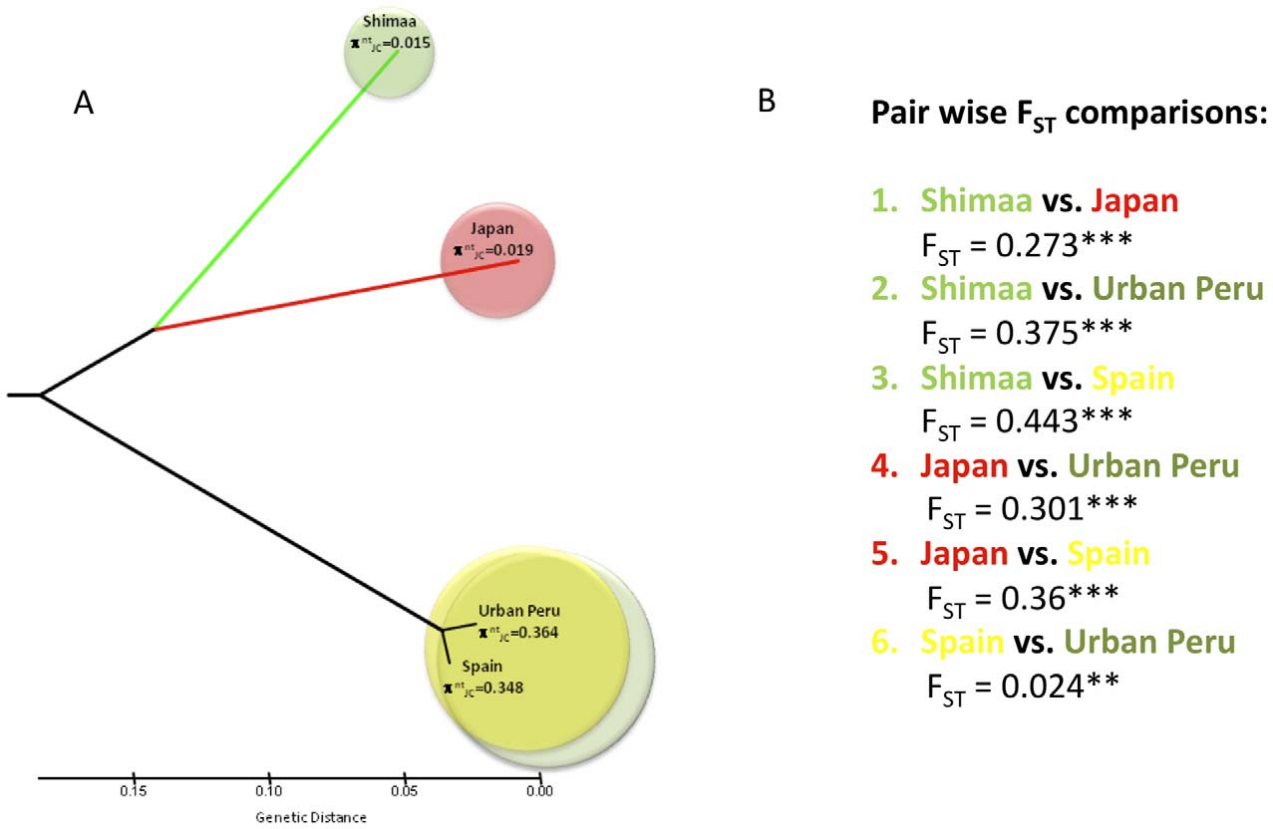
Genetic differentiation between *H. pylori* populations from Shimaa village, Japan, Spain and urban Peruvian shantytowns. **A.** An UPGMA tree was reconstructed using pairwise F_ST_ comparisons. F_ST_ values were calculated using sequences in a concatenated dataset of six housekeeping genes (∼3.5 Kb), as detailed in neighbor joining trees of Figs. S1, S2, S3, S4, S5, S6, S7. Circle diameters are proportional to the nucleotide diversity per site for each population. Bar scale  =  genetic distance measured in F_ST_ units. These analyses showed that Spanish, Japanese and Shimaa populations have diverged extensively, which we ascribe to geographic distance and a lack of gene flow between *H. pylori* populations. In contrast, no such extensive divergence between Spanish and urban (shantytown) Peruvian populations was detected; this is depicted with overlapping circles. **B.** Pairwise F_ST_ comparisons between populations. A low F_ST_ value, signifying lack of genetic differentiation, was seen only in Spanish vs. urban Peruvian strains. Asterisks indicate that all observed F_ST_ values were statistically significant, as determined by the permutation test done with 1000 replicates; see Tables S1, S2, S3, S4, S5, S6, S7, S8, S9 for details).

To obtain *H. pylori* likely to be of the purely Amerind type, strains were cultured from gastric biopsies from 44 residents of Shimaa, a 600-member village in the remote Peruvian Amazon. Analyses of concatenated sequences from six housekeeping genes placed each Shimaa strain in a discrete phylogenetic cluster, related to, but not intermingled with, strains from Japan. In contrast, the concatenated sequences from Peruvian shantytown strains were mostly intermingled with those from Spanish strains (Fig. 1A). The concatenated sequence data from individual strains are detailed in a neighbor-joining tree in Fig. S1. Trees generated using individual gene sequences (Figs. S2, S3, S4, S5, S6, S7) revealed a hybrid ancestry in 12 of the 33 shantytown strains, partly Amerind or Asian and partly European. On average ∼12% of alleles from shantytown strains seemed non-European (Figs S2, S3, S4, S5, S6, S7). This implies gene transfer and recombination between European and other lineages.

The distinctiveness of Shimaa strains is indicated quantitatively by relatively high values for F_ST_, the proportion of total genetic variance in a subpopulation relative to total genetic variance [31] (0.27–0.44) (Fig. 1B). This high F_ST_ indicates extensive genetic divergence, attributable to geographic isolation and a lack of gene flow between Shimaa and other populations. In contrast, a low F_ST_ value was obtained in comparison of urban Peruvian vs. Spanish strains (0.024), in accord with recent derivation of most shantytown and Spanish strain sequences from the same ancestral gene pool – i.e., the substantial displacement of Amerind by mostly European strains in urban Peruvians, noted above.

**Figure 2:**
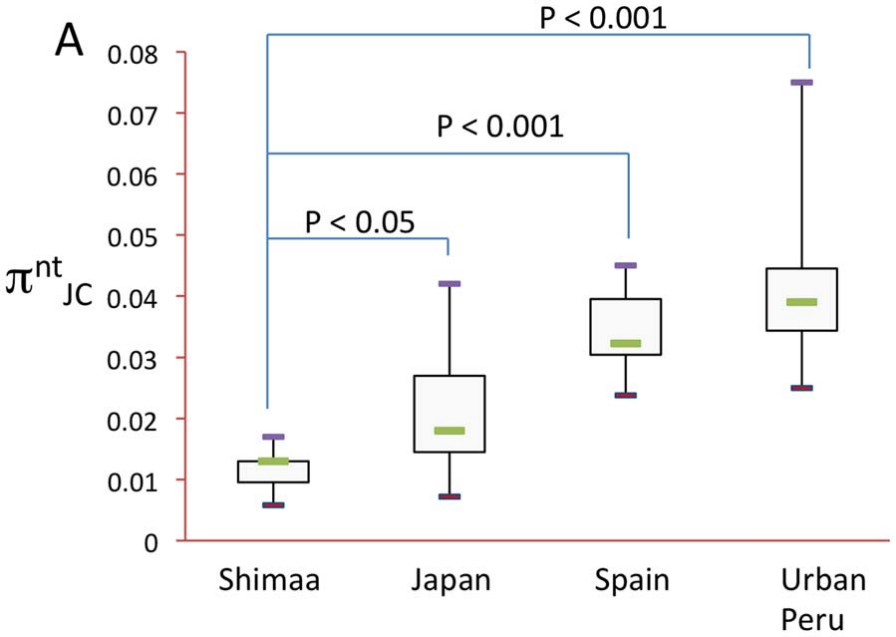
Differences among *H. pylori* populations in sequence diversity. The nucleotide (Nt) diversity per site (with Jukes-Cantor correction) was calculated for the six housekeeping genes (Fig. 1) and also for *hp0519* (Fig. 3), and is presented as a Box and Whisker plot for each population, showing the minimum, maximum, median and first and third quartiles. P-values were calculated using the T-test with 2-tails, assuming two samples with unequal variances.

Figs S2, S3, S4, S5, S6, S7 also show graphically that many Shimaa strains contain identical or nearly identical alleles of any given housekeeping gene, and that such identities are rare in *H. pylori* strains from larger, less isolated communities. Low Shimaa strain genetic diversity is further illustrated by median nucleotide sequence divergence per site (Fig. 2): 1.3% among Shimaa strains, vs. 1.8% among Japanese, and 3.2% and 3.9% in Spanish and urban Peruvian strains, respectively. The low diversity of Shimaa strains suggests a small effective population size (N_e_) [31], which could reflect relatively few founders, a low mutation rate, and/or the village's small size (only 600 people) and a resultant tendency to lose individual lineages.

### Colonization and virulence genes

PCR indicated that each of the 44 Shimaa strains contained an *s1* (potentially toxigenic) allele of the vacuolating cytotoxin (*vacA*) gene and a *cagA* gene. DNA sequencing identified two main clusters of alleles of the *vacA* middle region, the region that determines cell type specificity of toxin action [32]: 29 “*m1b*” type and *13* “*m2*” type; and also two “*m1b/m2*” recombinants (Fig. S8). Also found by PCR in each strain were genes *babA* and *sabA*, whose encoded proteins mediate adherence to the LewisB (branched fucose) and sialylated glycan receptors, respectively; and *babB*, which is *babA*-related but does not appear to mediate adherence [33], [34]. *babC* and *sabB* adhesin genes were not found in any Shimaa strain, and *hopZ*, which is implicated in adherence to cultured mammalian cells (receptor unknown) [35], was found in just 18 of the 44 Shimaa strains, not in the other 26.

**Figure 3:**
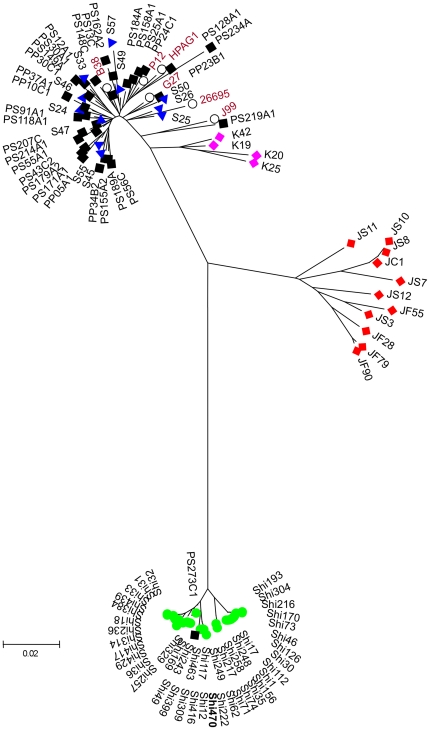
Neighbor joining phylogenetic tree of DNA sequences of gene *hp0519*. The full *hp0519* length (831 bp for most Shimaa strains) was used for each strain included in this tree. This tree shows massive separation of Shimaa *hp0519* alleles from those from elsewhere. The distinctiveness of Japanese alleles relative to Korean and European alleles had been documented previously [36]. The origins of *H. pylori* strains are coded by color and first letters of strain names: Shimaa, green (Shi); Japan, red (J); Korea, pink (K); Spain, blue (S); Peruvian shantytown, black (P). Genome sequenced reference strains from ethnic Europeans are indicated with unfilled circles (B38, P12, HpAG1, G27, 26695, J99). Bar scale indicates 0.02 nucleotide substitutions per site.

PCR and DNA sequencing indicated that the Shimaa alleles of gene *hp0519* (also postulated to affect host tissue structure or function [36]) differed markedly from those in other populations (≤72% and 86% amino acid and DNA sequence level identities, respectively). The majority of base substitution differences were non-synonymous (dN/dS  = 19.6) (Fig. 3), which implies a history of selection for changes in protein sequence. *hp0519* belongs to a multigene family whose encoded proteins are secreted and contain motifs resembling those found in “Sel1” eukaryotic regulatory proteins; the one family member examined to date, *hcpA*, was found to help regulate host immune responses to infection [36]–[38]. The ∼280 codon *hp0519* gene seems to have been fragmented in the genome-sequenced Venezuelan Amerindian strain v225d (genes *hpv225_0514* and *hpv225_0515*, 94 and 59 codons, respectively; Accession CP001582). Although *hp0519*'s biological role is not known, the divergence seen in Shimaa strains is reminiscent of that seen previously in Japanese strains (Fig. 3), which had been ascribed to adaptive evolution in the once-isolated Japanese island population [36]. Perhaps equivalent evolutionary forces operated on *hp0519* in isolated Amerindian populations.

### Transposable elements

Five members of the 2 kb IS*605* family have been found in *H. pylori* populations (IS*605* through IS*Hp609*), generally at frequencies that vary geographically [39]–[42]. PCR tests identified IS*607* in 32 of the 44 Shimaa strains and IS*Hp608* in 13 of them (each of which also contained IS*607*), but not the other three family members. Two IS*Hp608* variants are known: type 1, which is widespread in strains from Europe, Africa and South Asia; and type 2, found previously only in strains from The Americas (Peruvian shantytown, Alaska Native). IS*Hp608* seemed to be rare in or absent from East Asian *H. pylori* populations [41]. The Shimaa IS*Hp608* elements were type 2 (for sequence relationships, Fig. S9), further indicating that this element is a useful marker for Amerind *H. pylori* lineages. Also found in many Shimaa strains were “plasticity zone” (“TnPZ”) transposons [43], some of whose genes are virulence-associated in certain human populations [44], [45].

### DNA transformation

Representative Shimaa strains were tested for transformability with genomic DNAs from derivatives of strains 26695 and X47 that contained a *cat* (chloramphenicol resistance) gene in place of the non-essential *rdxA* nitroreductase gene [46]. Only two Cam^r^ transformant colonies were obtained from the five Shimaa strains tested (one each from strains Shi18 and Shi216), whereas >5,000 transformants were obtained in parallel using control strain X47 as a recipient. Furthermore no Shimaa strain transformants were obtained using genomic DNAs from 26695 derivatives containing *cat* in place of the *ureA-ureB* (urease) genes or an *aphA* (kanamycin resistance) gene in place of the *rdxA*-related *frxA* gene. Electroporation, which bypasses the need for early steps in competence was also attempted, with no better success. Analysis of Shi470's genome sequence (below) indicated that this strain contains all genes known to be needed for DNA transformation (Table S1) [47]. PCR tests of the few recovered transformants showed replacement of the resident intact *rdxA* gene by the Δ*rdxA-cat* allele, indicating homologous recombination, not *cat* insertion into an ectopic site.

We also tested if Shimaa strains could be transformed more efficiently with DNAs from closely related strains. First, genomic DNA from the Shi18 Δ*rdxA-cat* strain described above yielded ∼10,000 Cam^r^ transformants of its isogenic wild type parent and 60 Cam^r^ transformants of the distinct Shimaa village strain Shi470. Second, genomic DNA from a Shi470 Δ*rdxA-cat* transformant yielded ∼10,000 new Cam^r^ transformants of its isogenic wild type parent (*vs.* only ∼60 obtained using DNA from strain Shi18 Δ*rdxA-cat*). In another test, Shi470 was transformed efficiently with genomic DNA from a streptomycin resistant (*rpsL*-point mutant) derivative of strain 26695; ∼1,000 Str^r^ transformants were obtained. This high Str^r^ yield may reflect a need for only small patches of donor DNA (<100 bp) for point mutant allele transformation.

Thirty-one putative restriction modification systems were identified in the Shi470 genome sequence (see below), usually by specific methylase signatures, in accord with the great abundance of such gene clusters in other *H. pylori* strains (http://tools.neb.com/~vincze/genomes/). Some restriction-modification genes are strain-specific, and so these results suggest that restriction-modification systems of Shimaa strains could be functionally distinct from those of foreign strains, and could interfere with acquisition of gene sized DNA segments from them [48]. Alternatively, Shimaa strains might possess an aggressive DNA mismatch repair system that destroys incipient transformants made with divergent DNAs, much as is seen in *Salmonella*-*E. coli* crosses [49].

### Shimaa strain genome sequence

The features of Shimaa vs. Lima shantytown *H. pylori* described above suggested that European or hybrid (European-Amerindian, -Asian) strains were more fit than ancestral Amerindian strains. Given that multiple *H. pylori* strains from ethnic Europeans have been genome-sequenced, we elected to sequence the genome of a representative Shimaa village strain, Shi470, thereby to better evaluate the basis of fitness differences among strains and also gain more general insights into *H. pylori* genome evolution. Shi470 was cultured from an antrum biopsy of a 24-year old female with moderate chronic gastritis, mild to moderate glandular atrophy, and no detected intestinal metaplasia (Fig. S10). It was sequenced using 454 FLX technology, resulting in average read lengths of 276 bp, 71-fold coverage, and 50 large (>500 bp) contigs (Table 1). All contigs were connected and gaps filled (Materials and Methods). The Shi470 genome sequence was deposited in the NCBI database, annotated by NCBI pipeline staff (Accession CP001072), and released in May 2008.

**Table 1:** Shi470 genome sequencing raw statistics.

Parameter	Value
Average length of reads	276 bp
Coverage	71x
Number of contigs	65
Number of large contigs*	50
Number of bases	1,590,229 bp
Large contig bases	1,585,841 bp
Average size of large contigs	79,742 bp

* : a “large” contig is ≥500 bp long.

**Figure 4:**
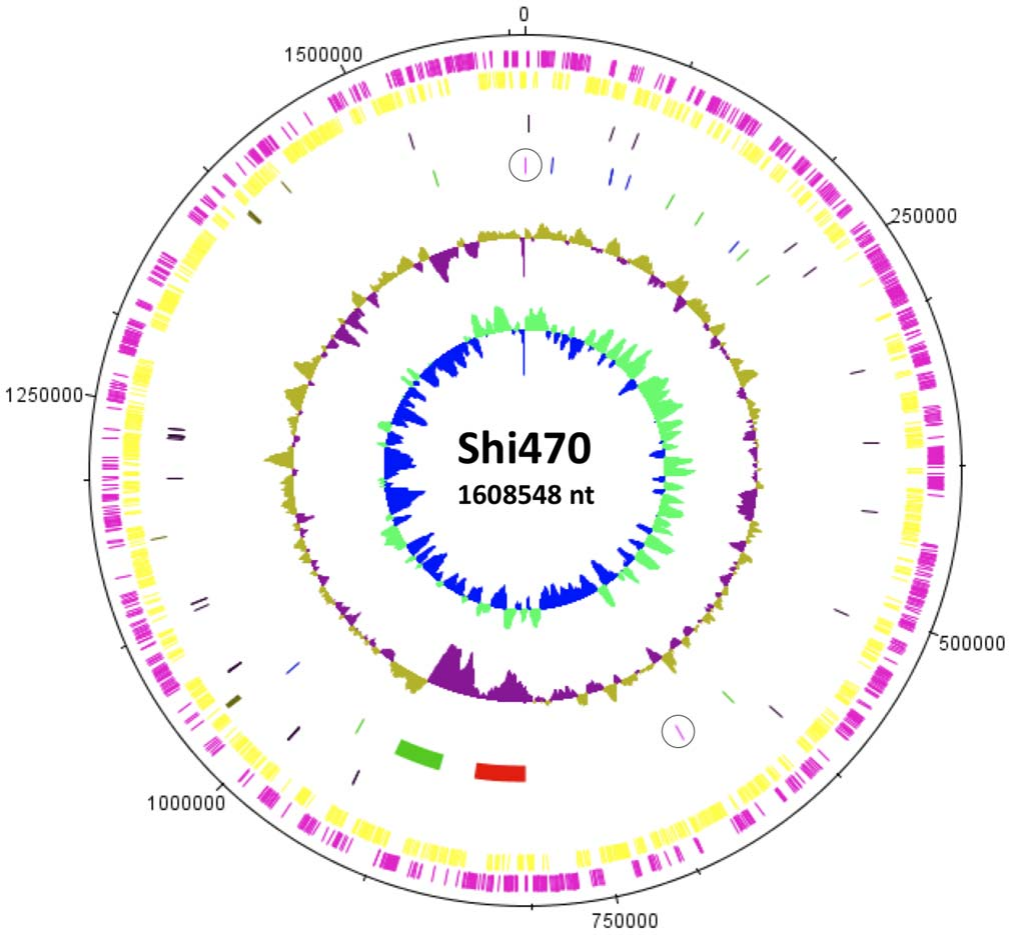
Organization of *H. pylori* strain Shi470 genome. The tracks from outside in represent: 1. Forward CDS (pink); 2. Reverse CDS (yellow); 3. rRNA (dark green); 4. tRNA (black); 5. Mobile elements: cag pathogenicity island (red bar), TnPZ plasticity zone transposon (green bar), mini IS*605* (green), and mini IS*606* (blue); 6. %GC plot (below and above average regions); and 7. GC skew [(GC)/(G+C)]. The locations of the replication origin (nt position 0) and terminus (*dif* site [50], near nt position ∼668325) are circled. The circular map was drawn using DNAPLOTTER (www.Sanger.ac.uk).

**Figure 5:**
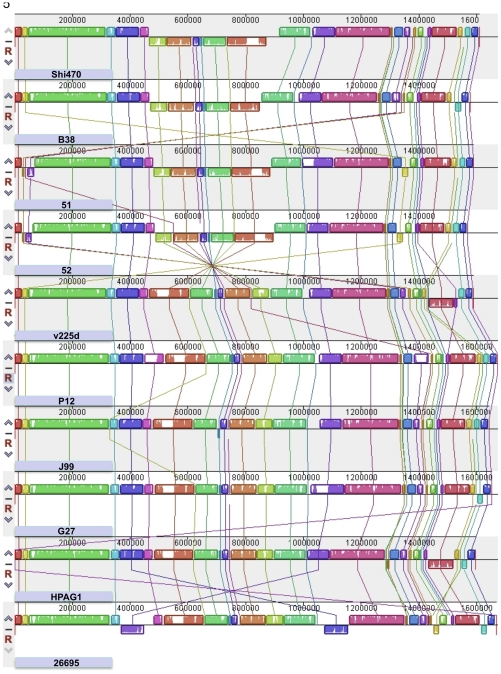
Comparison of chromosomal gene content and gene order in Shi470 and other sequenced *H. pylori* genomes. Complete chromosomal sequences of all *H. pylori* strains available in public databases by June 15, 2010 are compared using the Genome Alignment Visualization program (MAUVE; http://asap.ahabs.wisc.edu/mauve/). These strains, origins and genome accession numbers are: SHI470, Amerindian Shimaa villager, Peruvian Amazon, NC_010698; B38, France, NC_012973; 51, Korea CP000012.1; 52, Korea, CP001680.1; v225d, Amerindian, Piaroa, Venezuelan Amazon, CP001582; P12, Germany, NC_011498; J99, ethnic European, Tennessee USA, NC_000921; G27, Italy, NC_011333; HPAG1, Sweden, NC_008086; 26695, United Kingdom, NC_000915. Each horizontal panel contains a scale of strain genome sequence coordinates in base pairs, a series of colored blocks designating chromosome segments that aligned without internal rearrangement to segments in other strain chromosomes (connected by lines), and the strain designation. The relative orientations of DNA segments in the various strains are indicated by their positions above or below the genome center lines. Regions shown in white lack detectable homology among input chromosomes. The chromosomes of these ten strains show similar homology patterns, although with some rearrangements. The most prominent rearrangement involves a segment centered on the terminus of chromosome replication [50] that is in one orientation in Shi470 and three other strains, and in the reverse orientation in the other six strains. This segment is 450 kb long in Shi470, with endpoints likely to be in 108/111 bp inverted repeats between Shi470 nucleotide coordinates 465337–465447 and 915692–915582.

The Shi470 genome is plasmid-free and consists of a single circular chromosome, 1,608 kb in length (Fig. 4, Table 2). It is similar to other fully sequenced *H. pylori* chromosomes in size (range  = 1,569 kb–1,678 kb; see Fig. 5 legend), G+C content, and GC skew. Like many strains, it contains three clusters of Type IV secretion genes: one in the cag pathogenicity island (cag PAI), needed to deliver the CagA virulence protein and proinflammatory peptidoglycan fragments to host tissues [51], [52]; a second needed for DNA transformation [47]; and a third in the TnPZ transposon that is postulated to mediate DNA transfer during conjugation and/or delivery of effector proteins to host tissues [43]. Blastn and Blastx analyses identified only ∼3 kb that were present in the each of the other eight genomes that had been fully sequenced and released by May 2010 but that were absent from Shi470. Conversely only ∼5 kb (13 orfs) in Shi470 were absent from each of these other strains. These results imply that Amerind *H. pylori* have not undergone massive gene loss.

**Table 2:** General features of Shi470 genome sequence.

Feature	Value
Genome size (bp)	1,608,548
G+C content	38%
% coding	88%
Genes	1648
Protein coding	1569
Structural RNAs	42
23S-5S rRNA units	2
*vacA*	*s1b*, *m1b*
Genomic islands	*cag* PAI, TnPZ
IS elements	remnant IS*606*, miniIS*605*, miniIS*606*
Plasmids	none

BlastN, BlastX analyses of sequential 1 kb segments and neighbor joining phylogenetic tree construction also showed that nearly all of the Shi470 genome is more closely related to corresponding segments of Venezuelan Amazonian strain v225d and/or Korean strain genomes (51, 52) than to those of European strains (Only two 1 kb segments clustered more closely in neighbor joining trees with corresponding segments from any fully sequenced European strain than with v225d or 51 or 52; data not shown). Thus, we conclude that there has been little if any admixture of European type sequences in the Shi470 genome, despite occasional contacts between Shimaa villagers and people from elsewhere (e.g., health ministry personnel) – that the Shi470 genome is predominantly or entirely of the Amerind lineage. Similarly, we found that nearly all of the Venezuelan Amazonian strain v225d genome was more closely related to corresponding segments in Shi470 and/or Korean than European strain genomes.

Chromosome alignment showed a generally good conservation of overall gene order among *H. pylori* strains, although each individual strain could be distinguished from the others by a few small insertion/deletions (indels), and/or one or two larger rearrangements (Fig. 5). Most indels correspond to insertion sequences, restriction-modification genes and/or duplicate outer membrane protein genes or other repetitive DNAs. The most common major chromosome rearrangement involves a segment of some 450 kb in Shi470 that contains the terminus of chromosome replication, the *cag* PAI and this strain's TnPZ transposon. This segment is in the same orientation in three other strains (51 and 52 from Korea, B38 from France) and in the opposite orientation in six others including Venezuelan strain v225d (Fig. 5). Genome comparisons identified inverted repeats of 108/111 bp at the ends of this segment in Shi470, recombination in which would invert the segment relative to the rest of the chromosome. A further PCR test, however, indicated that this segment is in the same orientation in each of the 44 Shimaa strains, which implies that inversion is infrequent in this population.

### Shi470's cagPAI

**Figure 6:**
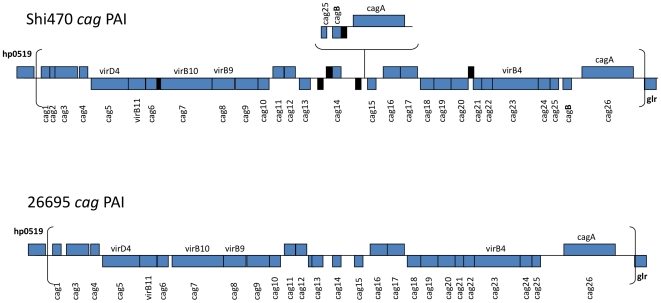
Gene duplication and translocation in Shi470 *cag* PAI. Shi470's *cag* PAI is similar to that of other *H. pylori* strains (here represented by 26695), except for a second copy of a segment containing a divergent *cagA* gene (*hpsh_04215*), *cagB* and part of *cag25* inserted between *cag*14 (*hpsh_04240*) and *cag*15 (*hpsh_04210*). The alleles of these *cagA* genes (*hpsh_04145*, normal location; *hpsh_04215*, transplaced copy) are more closely related to one another (81% and 83% protein level identities) than either is to *cagA* of strains from elsewhere (e.g, 79% and 77% protein level identities of *hpsh_04145* to *cagA* of Japanese strains, and European reference strain 26695. Similarly, the transplaced *cagA* gene *hpsh_04215* exhibits 71% and 68% protein level identities with *cagA* of representative Japanese strains and 26695. In contrast, the *cagB* alleles (*hpsh_4150*, *hpsh_4225*) exhibit 100% protein and 99% DNA level identities to each other. The NCBI pipeline had annotated five additional CDS in the Shi470 *cag* PAI (in black, *hpsh_04220*, *hpsh_4235*, *hpsh_4245*, *hpsh_4250* and *hpsh_4290* with sizes of 32, 31, 60, 45 and 44 codons). Each has DNA level homology with sequences that were considered to be intergenic regions in other strains. The DNA sequences of two (*hpsh_04235* and *hpsh_04250*) are well matched to sequences found in Japanese *cag* PAIs, whereas those of the others are matched to sequences found in *cag* PAIs in strains from around the world.

Shi470 contains a full set of *cag* pathogenicity island (PAI) genes with ∼95% average protein level identity (98% similarity) to those in reference strain *cag* PAIs, and also a second copy of a 4 kb, *cagA* and *cagB*-containing fragment inserted within the *cag* PAI (between *cag14* and *cag15*) (Fig. 6). PCR with flanking primers indicated that 14 of the other 43 Shimaa strains also contained this insertion; an equivalent *cagA-cagB* segment insertion was found in Venezuelan strain v225d [30]. Thus, this duplication/insertion may be widespread in Amazonian Amerindian strains, but it is not universal.

**Figure 7:**
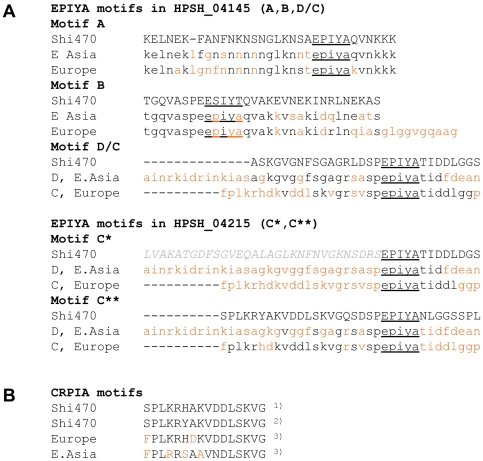
Host factor interaction motifs near C termini of Shi470 CagA proteins. **A.** Alignments of regions containing EPIYA (tyrosine phosphorylation) and CRPIA (conserved repeat responsible for phosphorylation independent activity) motifs in Shi470's two CagA proteins [products of *hpsh_04145* (normal position) and *hpsh_04215* (transplaced)]. Sequences from Shi470 proteins are compared with those of the most common prototype European and East Asian “A”, “B”, “C” and “D” EPIYA motifs described in [53]. **B.** The CRPIA motif sequences shown are from the following sources: ^1)^ The only CRPIA motif in *hpsh_04145* and the second of the two CRPIA motifs in *hpsh_04215*; ^2)^ The first of the two CRPIA motifs in *hpsh_04215*; ^3)^ Generic Western and East Asian motifs, as described [55], [56]. Black and orange, amino acid identity and divergence in Shi470 vs. most common Western or East Asian motifs, respectively. Segments designated motifs A, B and D/C are contiguous in CagA protein HPSH_04145. Similarly, segments designated C* and C** are contiguous in CagA protein HPSH_04215.

The proteins encoded by Shi470's two *cagA* genes differ markedly from one another (83% identity in 1059 shared positions) although they are more closely related to each other than to CagA proteins from other *H. pylori* strains. Of particular note are their tyrosine phosphorylation motifs (glutamic acid, proline, isoleucine, tyrosine, alanine; “EPIYA”), designated “A”, “B” and “C” or “D” in prototype CagA proteins based on flanking amino acid sequences, and also the nearby “dimerization” or “CRPIA” (“conserved repeat responsible for phosphorylation-independent activity”) motifs [53]–[56]. These various motifs interact with different constellations of cellular regulatory proteins, which, in turn, affect several competing regulatory subcircuits and epithelial tissue parameters. Shi470's normally placed *cagA* gene (*hpsh_04145*) encodes potentially functional A-like, degenerate B-like, and chimaeric D/C-like motifs, whereas the duplicate and transposed *cagA* (*hpsh_04215*) gene's product lacks A and B motifs, and contains two EPIYA motifs that each seem C-like although distinct from one another (Fig. 7). The two Shi470 CagA proteins also differ from prototype CagA proteins in their CRPIA motifs.

A direct repeat of 31 bp flanks the *cag* PAI in most *cag*+ strains, is present once at the “empty site” in strains that lack the PAI, and may serve as a recombination substrate for *cag PAI* insertion or excision. In Shi470, the *cag* PAI left end's repeat is replaced by a 110 bp remnant of IS*606*. This same replacement was also found in each of the other 43 Shimaa strains, which implies that the *cag* PAI should be stably maintained (not readily excised) in this population. Interestingly, all Shimaa strains contained motifs at the right end of the *cag* PAI of the type previously designated “III”, a type previously found to be abundant only in Indian *H. pylori* strains [25].

Shi470's vacuolating cytotoxin (*vacA*) gene (1287 codons) is of the *s1 m1* type, and most closely related to *vacA* of Venezuelan strain v225d, but diverges markedly from it near the site at which mature VacA protein is cleaved from the C terminal autotransporter segment (Shi470 residues 830–863) [32]. The generality of this divergence and its functional importance, if any, have not yet been tested.

### OMP families

Shi470 resembles other *H. pylori* strains in its large repertoire of outer membrane protein (*omp*) genes (Table S2). Prominent among them are the adhesin genes *babA* and *sabA*, which are specific for the LewisB (branched fucose) and inflammation-associated sialyl LewisX glycan receptors, respectively [33], [34]; and other genes also implicated in adherence to various target cells and tissues (although by as yet unknown mechanisms) (*hopZ*, *alpA*, *alpB*, *oipA* and *horB*) (Table S2). However, *sabA* and *hopZ*, and also *fecA2* and *frpB3*, which encode outer membrane proteins that contribute to uptake of iron or other essential metals, are pseudogenes in Shi470 – due, in each case, to nonsense or frameshift mutations. Missing from Shi470's repertoire are the adhesin-related *babC* and *sabB* genes, and *homA*, a member of the *hom* outer membrane protein gene family that is associated with benign infection in some populations [57].

### Remnant and vestigial IS elements

An IS*606* remnant (1376 bp of the ∼1967 bp element; lacking *orfA* transposase gene) occurs in Shi470 between the *ftsZ* gene (*hpsh_05170*, cell division) and an ion channel gene (*hpsh*_05200). This same remnant was found by PCR in 32 of the other 43 Shimaa village strains (and also in Venezuelan Amerindian strain v225d), in each case at the same location. In addition, six and eight copies of mini-IS*606* and mini-IS*605*, respectively, were found in the Shi470 genome – each containing some 100–150 bp from each end of the corresponding ∼2 kb full length elements (Fig. S11). The left end of each element was next to chromosomal sequences matching inferred target sites for insertion of full-length counterparts: 5′TTTAA or 5′TTTAAA for IS*605*, and 5′TTAT for IS*606* [39]. Each mini-IS element differed from others in the same group by some 10–20% in sequence, due to base substitution and small insertion/deletion mutation differences (Fig. S10). It is not known if any of these mini-IS elements have significant functional roles, e.g., through effects on expression of other chromosomal genes.

### Metronidazole (Mtz) resistance

Shi470 is Mtz resistant (forms colonies on agar with 32 µg Mtz/ml, in contrast to only ∼1-2 µg Mtz/ml for susceptible strains) [46]. Its *rdxA* (*hpsh_05025*) and *frxA* (*hpsh_03650*) nitroreductase genes, which are responsible for conversion of Mtz from prodrug to bactericidal agent, each contained null mutations, as is typical of Mtz^r^ strains [46]. Twenty of 39 other Shimaa village strains tested also were resistant to at least 16 µg Mtz/ml, and half of them contained nonsense, frameshift or deletion (null) *rdxA* gene mutations; other cases of resistance were likely due to missense mutations in these genes. The frequent occurrence of resistance to Mtz in Shimaa strains may reflect sporadic provision of this drug to villagers by the Peruvian Health Ministry for use against parasitic infections and other illnesses.

## Discussion

The Asian-related sequences of Shimaa village *H. pylori* (Fig. 1; Figs S1, S2, S3, S4, S5, S6, S7) suggest that these bacteria descend from strains of the ancestral Amerindians who migrated into The Americas some 15,000 or more years ago. Although residents of Lima shantytowns are also of predominantly Amerindian ancestry their *H. pylori* strains seem mostly European, with some Amerind and/or Asian admixture. This suggests displacement of original Amerind type strains by predominantly European or hybrid strains, presumably because they were more fit. Displacement of ancestral Amerind strain types was also invoked to explain similar results from studies of *H. pylori* from Venezuela and Colombia [30].

Just a few alleles of any given gene predominated among Shimaa *H. pylori* strains. This contrasts with the rarity of identical alleles in independent isolates from most other populations (illustrated in Fig. S2, S3, S4, S5, S6, S7, S8, S9) [15], [16]. The Shimaa strains' low genetic diversity can be ascribed to (i) descent from small numbers of ancestral *H. pylori* lineages, due in turn to the relatively few people who migrated to Beringia and ultimately into The Amazon long ago [23]; (ii) the small size and remoteness of Shimaa village; and (iii) conditions that facilitate *H. pylori* transmission between households and sporadic loss (replacement) of individual strains [5], [6], [12], [13].

It is with this background that we sequenced the genome of Shimaa village strain Shi470. Our analyses indicate that it is quite purely Amerindian, that all but possibly a few kb of its 1.6 mb genome are more closely related to corresponding sequences in the genomes of the other available Amerindian strain genome (v225d) and/or the two Korean strains (51, 52) than to those of strains from ethnic Europeans. We conclude that Shi470, and also the Venezuelan Amazonian strain v225d, are of the ancient Amerind lineage, and anticipate that their two genome sequences should be a valuable resource for further analyses of *H. pylori* genome evolution in Native peoples of The Americas. More generally, all sequenced *H. pylori* genomes seem similar in their content of conserved and strain-specific genes (Fig. 5). This indicates that perceived lower fitness of Amerind strains is not likely to be due to wholesale gene loss during the millennia that they were isolated from those of Eurasia and Africa. Shi470 contains the two most prominent DNA segments found to be strain-specific, to be missing from significant numbers of strains in at least some populations: (i) the disease-associated cag pathogenicity island [53], [54]; and (ii) a TnPZ (plasticity zone) transposon, some of whose genes also have been implicated in virulence [44], [45]. Also prominent in Shi470 is a 450 kb segment that contains the terminus of chromosome replication and that is likely to be invertible: this segment is in Shi470's orientation in three other genome-sequenced strains, and in the opposite orientation in six others, including Amerind strain v225d. Inverted repeats of ∼100–200 bp at this segment's endpoints suggest that inversion could occur by RecA-mediated homologous recombination, but the uniformity of this segment's orientation among Shimaa strains suggests that inversion is rare, at least in this population.

Multiple genetic determinants are each likely to contribute to the apparent difference in fitness between Amerind and European or hybrid strains, as is the case with many quantitative traits in diverse organisms [58]. Among likely contributors are genes whose products interact directly with host cells. In particular, Shi470's two CagA proteins are unusual in their sets of EPIYA tyrosine phosphorylation and CRPIA motifs, which likely affect cytoskeletal and tissue structure, the induction of proliferative and proinflammatory responses, and the potency of *H. pylori*'s VacA cytotoxin (and thereby, potentially VacA-regulated traits such as tissue leakiness, apoptosis, and immune responses) [32], [59]–[61]. This possibility has also been discussed in studies of Amerindian strain v225d [30]. Far less is known about *hp0519*, a member of a multigene family whose encoded and secreted proteins contain motifs characteristic of the Sel1 family of eukaryotic regulatory proteins. However, Shimaa strain *hp0519* alleles are also highly divergent from those of other populations, with most DNA sequence differences affecting the encoded protein's amino acid sequence (dN/dS  = 19.6) (Fig. 6). This is reminiscent of the intense selection for amino acid change seen previously in alleles from the Japanese islands, and which was ascribed to selection for adaptation to local conditions [34]. Future studies will test if Hp0519 protein, like at least one other Sel1-like family protein (HcpA [37]), interacts with a host component during infection; and also if the strength or specificity of this interaction is affected by sequences that distinguish the Shimaa Hp0519 proteins from those of other populations.

One formal explanation for the proposed substantial displacement of Amerind by European or hybrid strains invokes differences in direct competitive ability. For example, Amerind strains might have lost vigor due to genetic drift (chance mutation, fortuitous fixation of deleterious alleles) during migrations of small founder populations from Asia into Beringia and ultimately into the Americas [23]. Or, less vigorous strains might have been less debilitating to their hosts during their migrations or residence in harsh (e.g. Arctic) environments; those that least impaired human survival would have enjoyed the best chances of transmission from adults to their infants, and thus persistence in these small human populations. In accord with these lower in vivo fitness explanations, Shimaa village strains grew more slowly than most shantytown isolates under our standard in vitro culture conditions (BHI blood agar; microaerobic atmosphere). In either explanation, the apparently low efficiency of transformation of Shimaa strains with DNAs from unrelated strains might reflect a relative inability to acquire foreign (e.g., European) DNA during human infection, which, in turn, might make Amerindian strains less able than European strains to adapt to variable host conditions, and thus less fit. This scenario would explain why European type sequences predominate in most strains from Lima shantytown residents. A third explanation emerges from the idea [9], [10], although controversial [11], that some *H. pylori* infections may be beneficial; indications that *H. pylori* infection can affect innate immune responses [62], [63]; and at least partial protection by innate immune mechanisms against many viral infections [64], [65]. We can imagine that the types of innate immune responses stimulated by European or hybrid strains contributed more effectively than did the responses elicited by purely Amerind type strains to human survival – variously during the new epidemics that accompanied the European conquest, or in modern urban shantytowns.

In conclusion, the characteristics of Amerind strains from the remote Amazonian village, Shimaa, suggest descent from strains carried to the Americas by ancestral Amerindians many thousands of years ago, and substantial displacement by strains of European or hybrid ancestry. The distinctive features we found in Amerindian strain Shi470 include novel alleles of the *cagA* virulence gene and *hp0519*, genes that may each affect bacterial-host interactions. Hypotheses for the displacement of Amerind by European strains that merit testing include differences in fitness per se, vs. selection for *H. pylori* genotypes that contribute to human host survival, variously during ancestral migrations, during the colonial period or in modern shantytowns.

## Materials and Methods

### Ethics Statement

Forty-four *H. pylori* strains studied here were cultured in May 2006 from gastric biopsy specimens from residents of the village of Shimaa in the remote Peruvian Amazon who were symptomatic and had accepted an offer of diagnostic endoscopy, as also described in ref. [43]. Endoscopy was preceded by explanations and discussions of the procedure, risks and anticipated uses of the biopsies – first with the village chief, and then with villagers. These discussions and explanations were carried out in Spanish, and also in the native Machiguenga language of this village, with the aid of a Spanish-Machiguenga interpreter, and in the presence of their trusted physician (in residence for two years) from the Peruvian Ministry of Health. Strains from Lima region shanty towns (San Juan de Miraflores and Puente Piedra) were similarly cultured from gastric biopsies, also obtained after equivalent discussions in Spanish. All endoscopies were performed with informed consent (written or verbal, depending on participant's literacy) for bacterial culture and genetic analyses, as described here, under protocols approved by the Human Studies Committees of Johns Hopkins University (Baltimore, MD, USA), of AB Prisma and of Universidad Peruana Cayetano Heredia (Lima, Peru). These three institutional review board committees had, in particular, approved the endoscopy procedure, the written and verbal informed consent procedures, and the bacterial culture and genetic (DNA sequence) analysis experiments. Other *H. pylori* strains used here were from the Berg lab collection, and had been kindly provided by Drs. Teresa Alarcon and Manuel Lopez Brea (Spain) and Teruko Nakazawa (Japan) from their collections.

### General bacteriologic, molecular and histologic methods

*H. pylori* was grown on brain heart infusion agar (Difco) containing 7% horse blood and 0.4% isovitalex in a microaerobic (5% O_2_, 10% CO_2_) atmosphere following standard protocols [25], [43]. Chromosomal DNA for genome sequencing and routine PCR was isolated using the QIAamp DNA Mini kit (Qiagen, Chatsworth, CA). Genomic DNA of higher molecular weight, needed for efficient direct chromosomal sequencing was isolated using hexadecyltrimethylammonium [40]. PCR amplification, product purification, and capillary DNA sequencing, both of PCR products, and directly from chromosomal DNA, were carried out as described [42], [43]. DNA sequence editing and analysis were performed with programs in Vector NTI (Informax, Bethesda, MD); programs and data in *H. pylori* genome sequence databases, and Blast homology search programs (http://www.ncbi.nlm.nih.gov/blast/blast.cgi). Unrooted trees were constructed by Neighbor-Joining (Mega 3.1, http://www.megasoftware.net/). Gastric pathology was scored on antrum and corpus biopsies that had been fixed in pH 7.2 buffered formalin, embedded in paraffin, sectioned, stained with hematoxylin/eosin and graded histologically as described [66].

### Genome sequencing

Genomic DNA prepared using a Qiagen kit from a low passage single colony isolate was sequenced using 454 FLX technology by the 454 Corporation. An average read length of 276 nts and 71x coverage was achieved. The sequences were arranged in 50 major contigs of at least 500 bp (average 79.7 kb). Contigs were aligned using the fully sequenced J99 and 26695 reference genomes. Closure of gaps and connection of contigs into the final finished genome sequence was done manually by PCR to identify connections, capillary sequencing PCR products and directly from genomic DNAs [43]. Approximately 20 kb of additional sequence were determined in this way. Genome annotation was carried out by NCBI using their Automated Pipeline (http://www.ncbi.nlm.nih.gov/Genbank/genomesubmit.html). Left unchanged was the Pipeline gene annotation format, in which sequential orfs were counted in fives, to allow later insertion of additional orfs in series when needed. Eight sites of frameshifts in repetitive sequences (where 454 technology is most prone to base counting errors) in genes of known function were resequenced manually. In only one of the eight cases was an error found, and this manual resequencing allowed restoration of the gene to its active form. These operations resulted in the single circular genome sequence depicted in Fig. 3 and reported in NCBI accession CP001072. Table 1 summarizes the raw sequencing statistics.

### DNA sequence analysis and comparison

Genomic DNA sequences were analyzed using Personal Blast Navigator PLAN (http://bioinfo.noble.org/plan/), Genome Alignment Visualization MAUVE 2.2.0 (http://asap.ahabs.wisc.edu/mauve/, Genome Evolution Laboratory, Genome Center of Wisconsin) and NCBI BLAST (http://blast.ncbi.nlm.nih.gov/Blast.cgi). Neighbor Joining trees of *H. pylori* from selected populations were created by Molecular Evolutionary Genetics Analysis (MEGA, version 3.1; http://www.megasoftware.net/); the DNA sequence polymorphism (DnaSP, http://www.ub.es/dnasp/) program was used to convert sequences from Fasta to Mega format.

### GenBank Accessions

Individual gene sequences were PCR amplified and sequenced with primers listed in Table S3. The GenBank Accession numbers of DNAs from Shimaa and other *H. pylori* populations sequenced specifically for this study are as follows: *atpA*, GU045831-GU045915; *cysS*, GU045916-GU045987; *glm*, GU045988-GU046066; *glr* (*murI*), GU046067-GU046139; *ppa*, GU046140-GU046225; *recA*, GU046226-GU046307; *hp0519*, GU064397-GU064440; *vacAm1*, GU064441- GU064486; *vacAm2*, GU064487- GU064499; *vacAs1*, GU064500-GU064527; and IS*607*, GU064528-GU064554. Other sequences used in to determine genetic relatedness of *H. pylori* from Shimaa and Lima shantytown residents described here are found in GenBank under the strain names shown in Figs. S1, S2, S3, S4, S5, S6, S7 (Spanish (“S”) strains are listed with a “HUP-B” prefix; and Peruvian strains with a single “P” prefix are designated “PS” in GenBank).

## Supporting Information

Figure S1 Neighbor-Joining tree of concatenated sequences from six housekeeping genes.
*H. pylori* from four populations were analyzed: remote Peruvian Amazon village of Shimaa (44 strains, in green circles), Japan (18 strains, in red diamonds), Spain (20 strains, in blue triangles) and from Amerindians from shantytowns in urban (Lima) Peru (18 strains, in black squares) were compared by concatenated evolutionary tree of six housekeeping genes (3354 bp in total): *atpA* (849 bp), *recA* (606 bp), *glmM* (*ureC*, 555 bp), *ppa* (339 bp), *cysS* (504 bp) and *glr* (*murI*) (501 bp). Arrow designates Shi470, whose complete genome sequence is reported here. Open circles identify sequences from other reference fully sequenced genomes (v225d, Venezuela (Amerindian); 51, Korea; 52, Korea; 26695, UK; G27, Italy; HPAG1, Sweden; J99, US (Caucasian); B38, France).
(TIF)

Figure S2 Neighbor joining tree of sequences from *glmM* gene (strain 26695 *hp0075* homolog). Color coding as in Fig. S1
(TIF)

Figure S3 Neighbor joining tree of sequences from *recA* gene (strain 26695 *hp0153* homolog). Color coding as in Fig. S1
(TIF)

Figure S4 Neighbor joining tree of sequences from *glr* (*murI*) gene (strain 26695 *hp0549* homolog). Color coding as in Fig. S1
(TIF)

Figure S5 Neighbor joining tree of sequences from *ppa* gene (strain 26695 *hp0620* homolog). Color coding as in Fig. S1
(TIF)

Figure S6 Neighbor joining tree of sequences from *cysA* gene (strain 26695 *hp0886* homolog). Color coding as in Fig. S1
(TIF)

Figure S7 Neighbor joining tree of sequences from *atpA* gene (strain 26695 *hp1134* homolog). Color coding as in Fig. S1
(TIF)

Figure S8 Neighbor joining tree of the *vacA* gene mid region, which determines cell type specificity of VacA toxin action. This shows that Shimaa *vacA* alleles are most related to but distinct from those of Japan, and that some Peruvian shantytown strain *vacA m1* alleles are closely related to those of Shimaa strains whereas others are intermingled with those from Spain; and that Shimaa *vacA m2* alleles are related to but distinct from those of Okinawa (few if any *vacA m2* alleles have been found in Japanese main island or Peruvian shantytown strains).
(TIF)

Figure S9 Neighbor joining tree of IS*607* and IS*Hp608* sequences found in Shimaa vs. other strains. The IS*607* tree was generated from a central 770 bp segment containing 146 of the *orfA* transposase gene's 217 codons, 71 of accessory gene *orfB*'s 419 codons. Similarly, the IS*Hp608* tree was generated from a 654 bp segment containing 88 codons of the 155 codon *orfA* transposase gene and 101 codons from the 382 codon *orfB* gene.
(TIF)

Figure S10 Hematoxylin and eosin stained antrum biopsy sections of Shi470 infected and uninfected Peruvians.
A. Gastric biopsy from antrum of Shimaa villager naturally infected with Shi470. Evident here are chronic active antral gastritis with moderate activity (multiple polymorphic neutrophils seen at higher magnification) and moderate chronic inflammation (I) of the lamina propria (LP) extending down to muscularis mucosa (M). Moderate hyperplasia of epithelial cells is seen along the columnar epithelium (E) extending throughout the gastric pits (P). There is moderate glandular atrophy (A) with partial replacement of deep glands with fibrous tissue in areas where the gastric glands (G) should be extending down to the muscularis mucosa. A primary lymphoid follicle is also present as seen by the spherical mass of chronic inflammatory cells (F). Glandular secretions are seen along epithelial surface (X).
B. Antrum biopsy section of uninfected antrum from Lima resident. Seen here is uninfected gastric mucosa with columnar epithelial cells (E) and supporting lamina propria (LP) extending down to the start of the muscularis mucosa. The lamina propria of this individual is populated primarily with mesenchymal cells and a few sparse lymphocytes. The stomach antrum contains tightly packed branching tubular glands that open up into irregularly shaped gastric pits (P). The mucus secreting cells of the deep glands play a role in protecting the intestinal mucosa. Note that these glands (G) extend the entirety of the gastric mucosa reaching to the muscularis mucosa at their deepest point. Glandular secretions are seen along the epithelial surface (X).
(TIF)

Figure S11 Sequence alignment of mini-IS*605* and mini-IS*606* elements found in Shi470 genome, relative to those in reference strains. Chromosomal sequences adjacent to mini IS element left ends, positions of left end, and mini-IS orientation [clockwise (c) or counter clockwise (cc)] are indicated.
(TIF)

Table S1 Shi470 genes involved in natural transformation
(PDF)

Table S2 Outer membrane protein genes in Shi470
(PDF)

Table S3 Primers used for analysis of Shimaa village strains
(PDF)
