# Supplementary figures and images for: Helicobacter pylori from Peruvian Amerindians: Traces of Human Migrations in Strains from Remote Amazon, and Genome Sequence of an Amerind Strain
Source: PLoS One. 2010 Nov 29;5(11):e15076. doi: 10.1371/journal.pone.0015076 (PMC2993954; doi:10.1371/journal.pone.0015076)

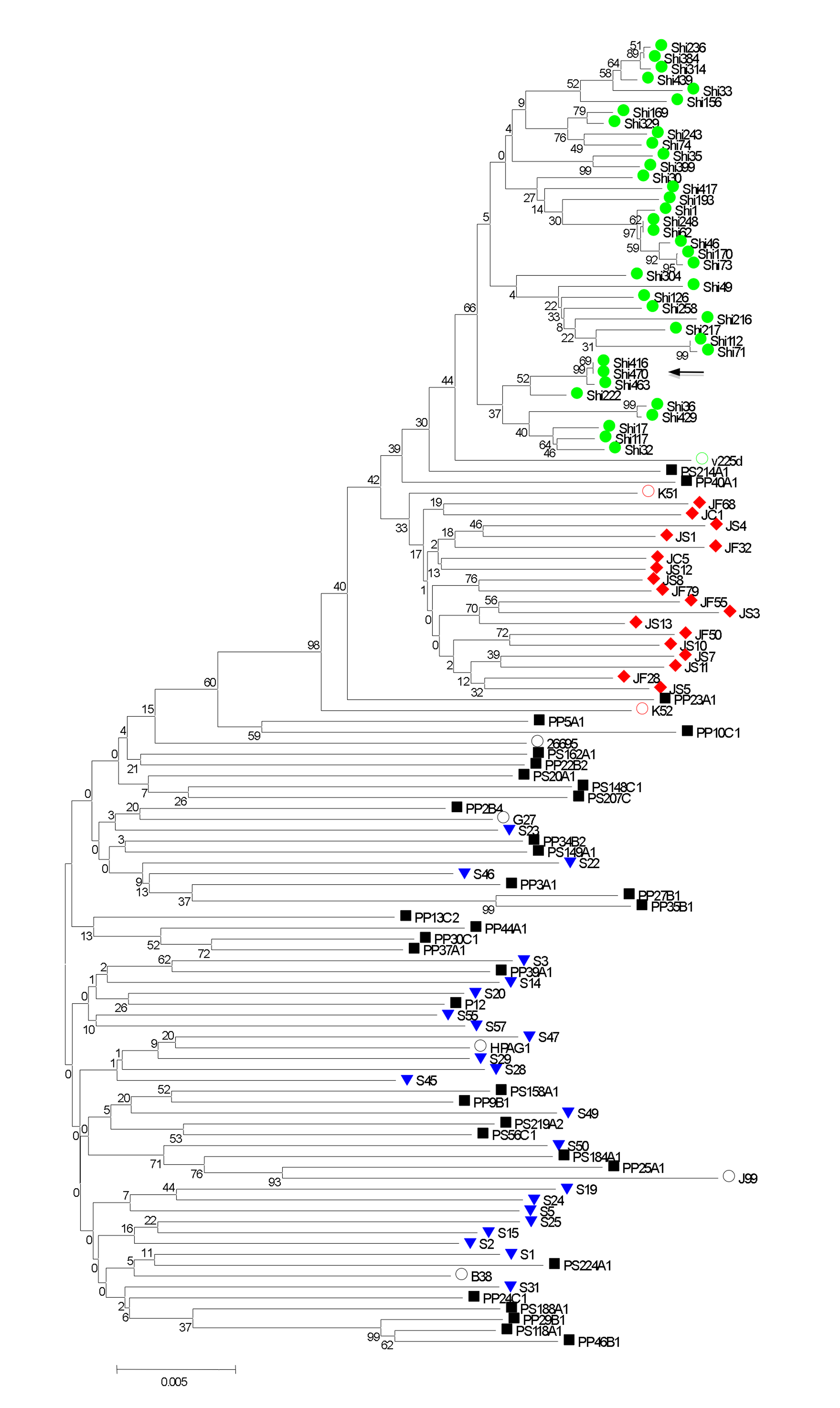

Supplement: Figure S1 — Neighbor-Joining tree of concatenated sequences from six housekeeping genes. H. pylori from four populations were analyzed: remote Peruvian Amazon village of Shimaa (44 strains, in green circles), Japan (18 strains, in red diamonds), Spain (20 strains, in blue triangles) and from Amerindians from shantytowns in urban (Lima) Peru (18 strains, in black squares) were compared by concatenated evolutionary tree of six housekeeping genes (3354 bp in total): atpA (849 bp), recA (606 bp), glmM (ureC, 555 bp), ppa (339 bp), cysS (504 bp) and glr (murI) (501 bp). Arrow designates Shi470, whose complete genome sequence is reported here. Open circles identify sequences from other reference fully sequenced genomes (v225d, Venezuela (Amerindian); 51, Korea; 52, Korea; 26695, UK; G27, Italy; HPAG1, Sweden; J99, US (Caucasian); B38, France). (TIF) [file pone.0015076.s001.tif]

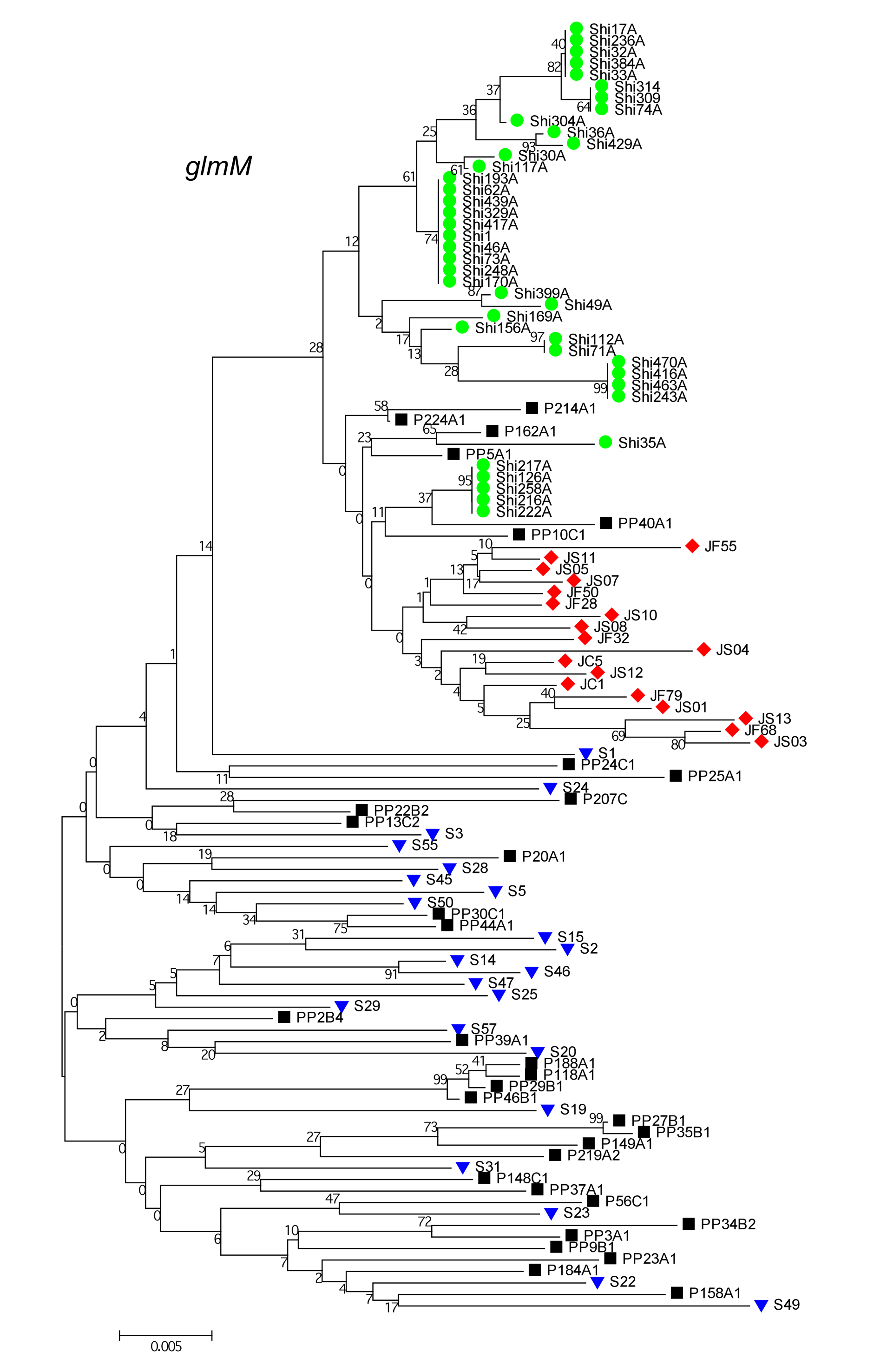

Supplement: Figure S2 — Neighbor joining tree of sequences from glmM gene (strain 26695 hp0075 homolog). Color coding as in Fig. S1 (TIF) [file pone.0015076.s002.tif]

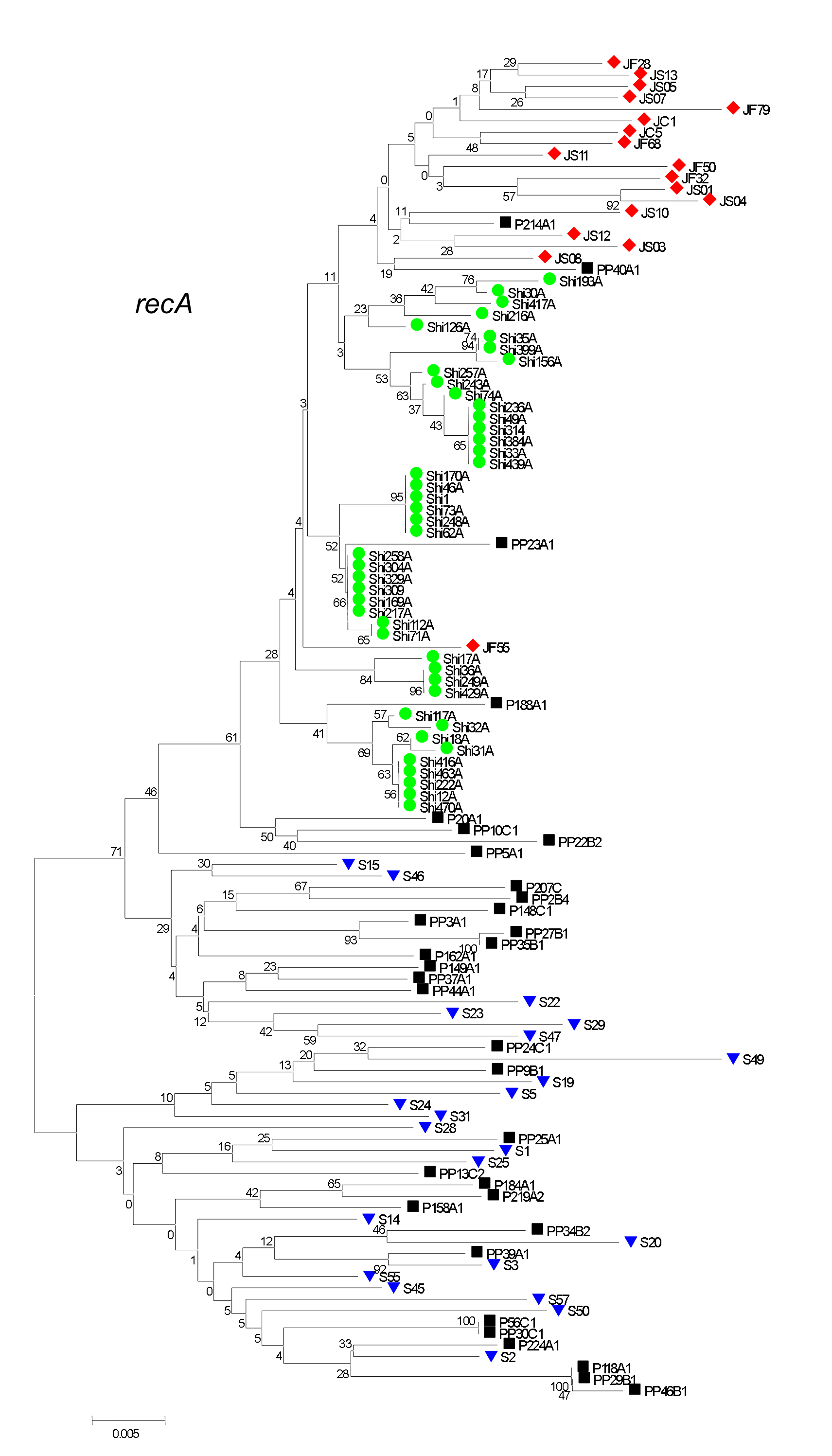

Supplement: Figure S3 — Neighbor joining tree of sequences from recA gene (strain 26695 hp0153 homolog). Color coding as in Fig. S1 (TIF) [file pone.0015076.s003.tif]

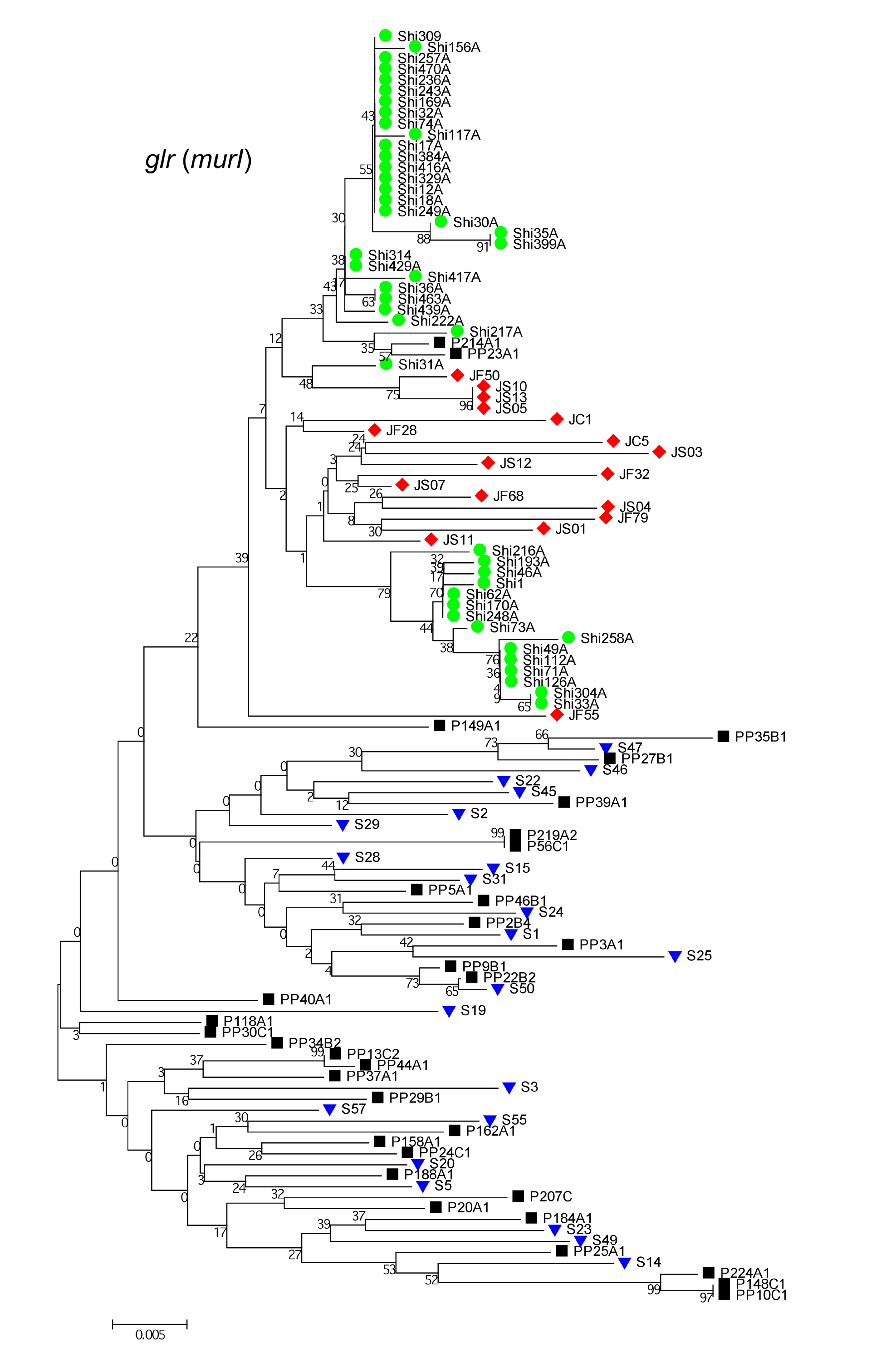

Supplement: Figure S4 — Neighbor joining tree of sequences from glr (murI) gene (strain 26695 hp0549 homolog). Color coding as in Fig. S1 (TIF) [file pone.0015076.s004.tif]

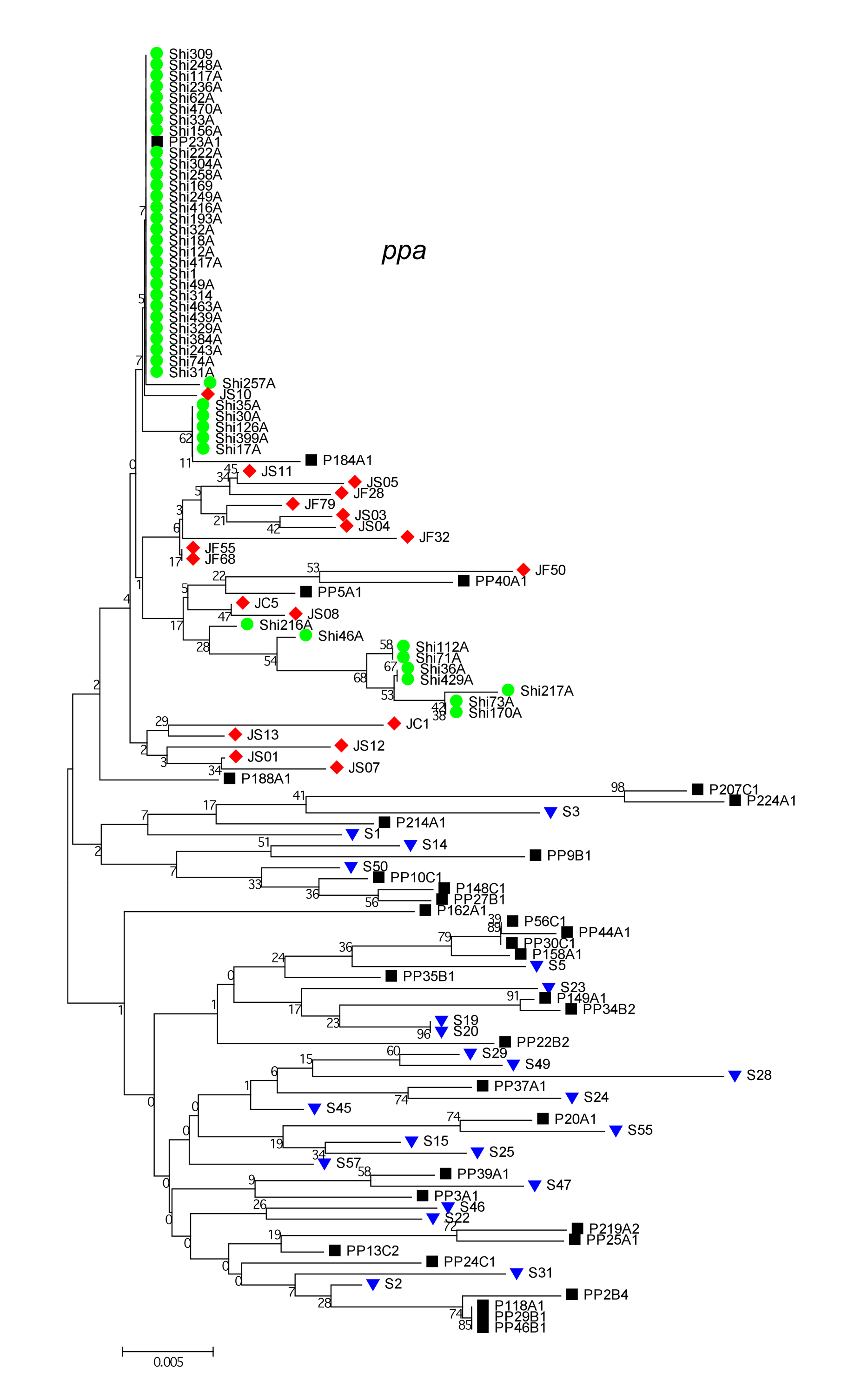

Supplement: Figure S5 — Neighbor joining tree of sequences from ppa gene (strain 26695 hp0620 homolog). Color coding as in Fig. S1 (TIF) [file pone.0015076.s005.tif]

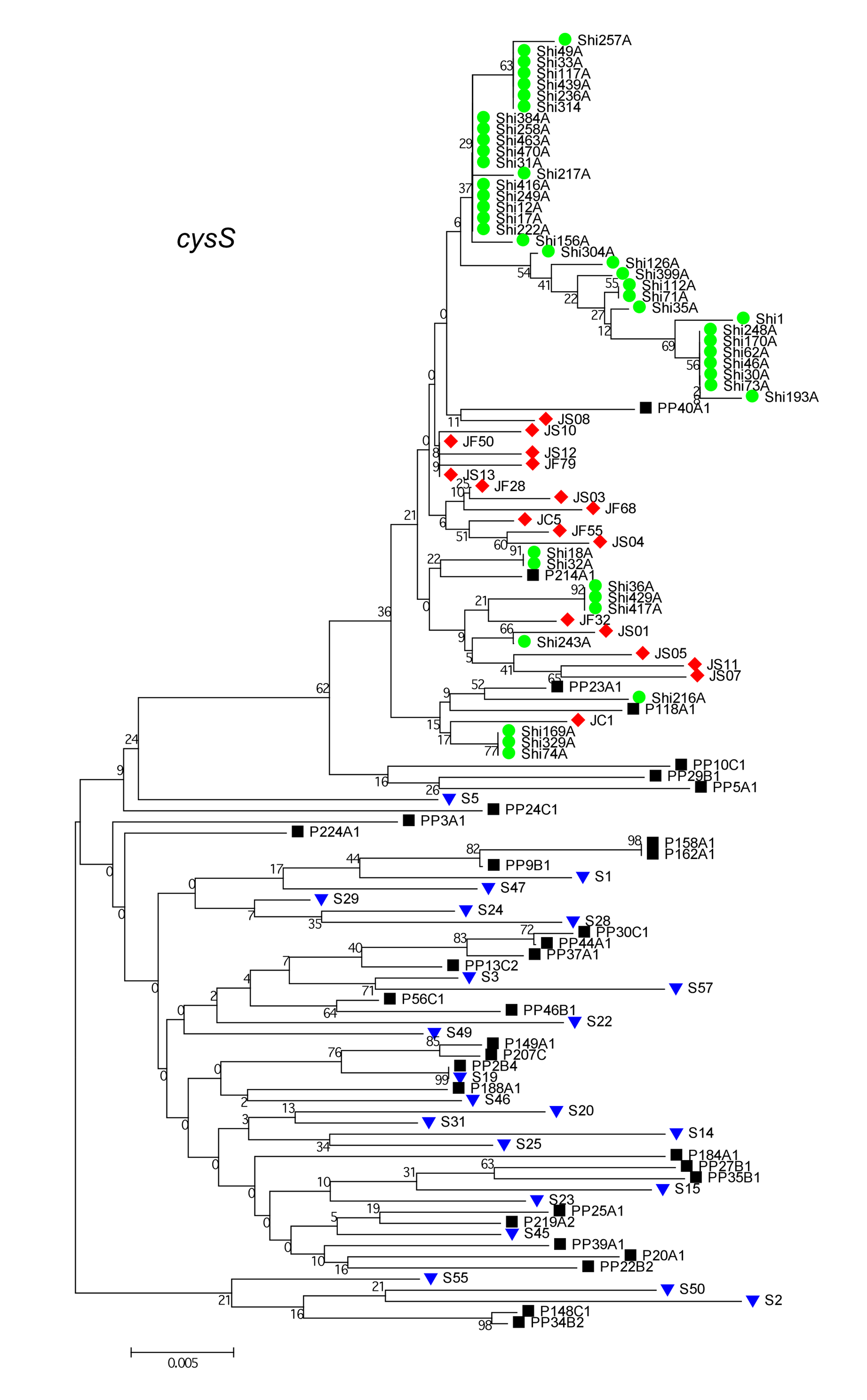

Supplement: Figure S6 — Neighbor joining tree of sequences from cysA gene (strain 26695 hp0886 homolog). Color coding as in Fig. S1 (TIF) [file pone.0015076.s006.tif]

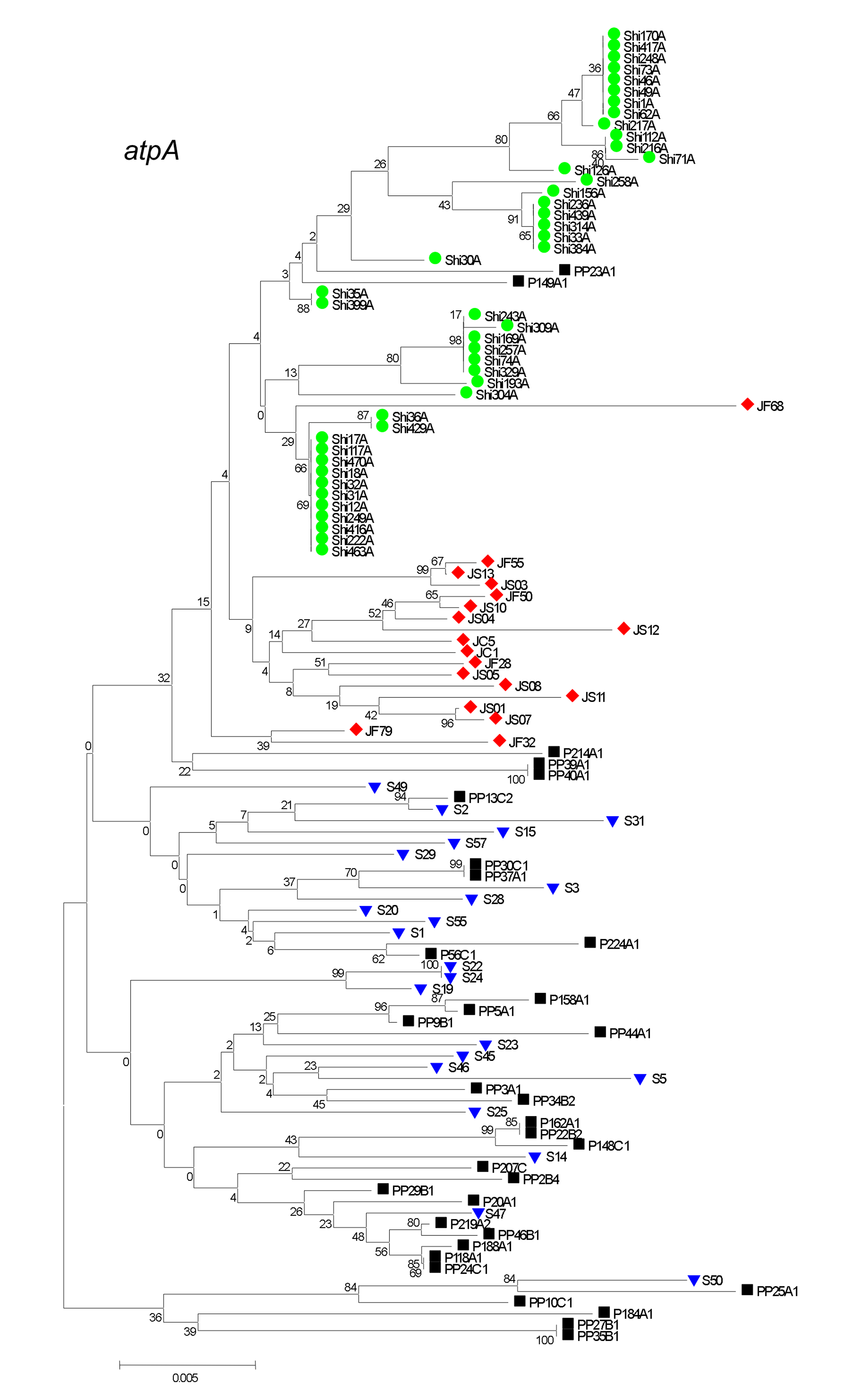

Supplement: Figure S7 — Neighbor joining tree of sequences from atpA gene (strain 26695 hp1134 homolog). Color coding as in Fig. S1 (TIF) [file pone.0015076.s007.tif]

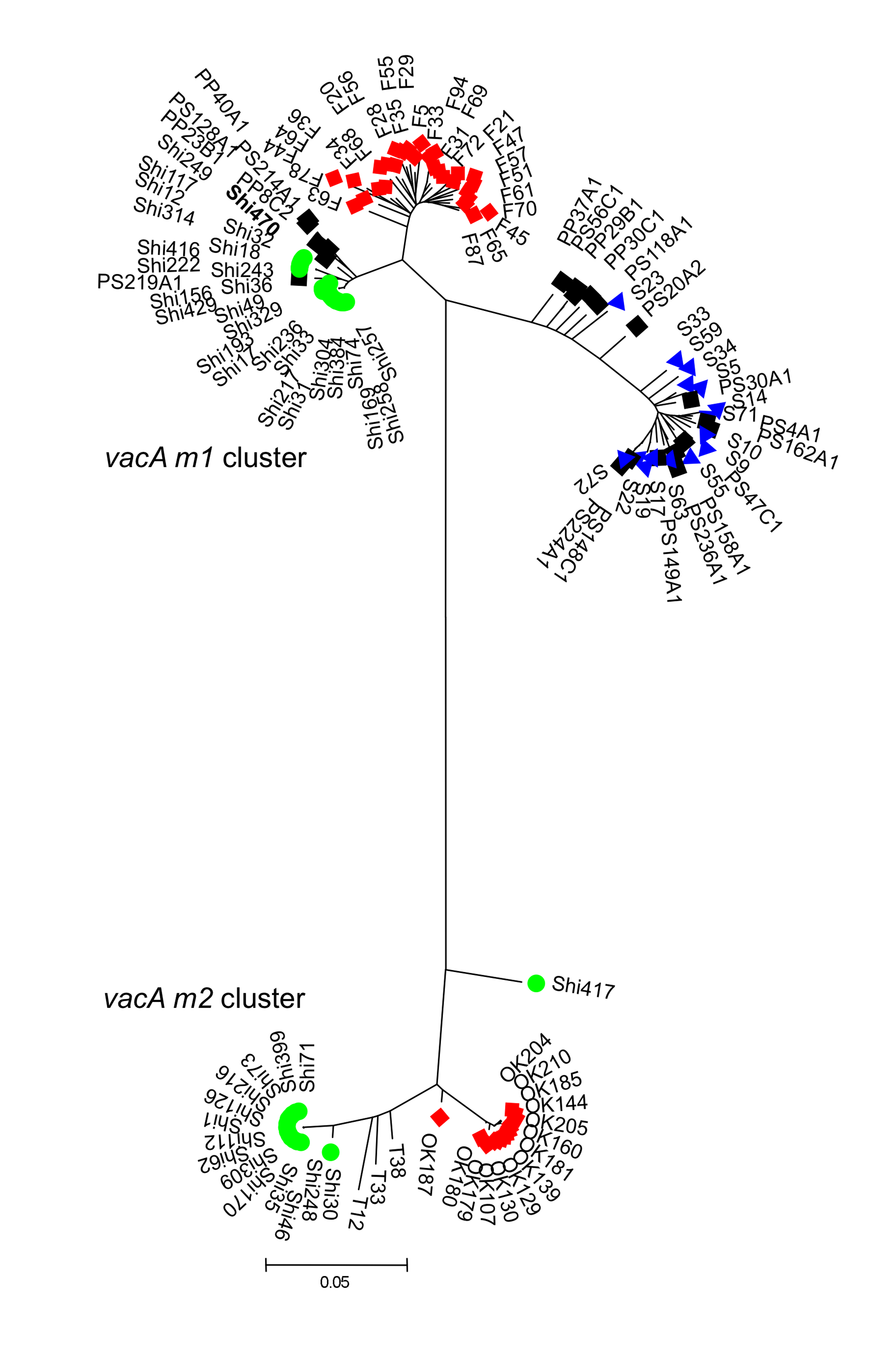

Supplement: Figure S8 — Neighbor joining tree of the vacA gene mid region, which determines cell type specificity of VacA toxin action. This shows that Shimaa vacA alleles are most related to but distinct from those of Japan, and that some Peruvian shantytown strain vacA m1 alleles are closely related to those of Shimaa strains whereas others are intermingled with those from Spain; and that Shimaa vacA m2 alleles are related to but distinct from those of Okinawa (few if any vacA m2 alleles have been found in Japanese main island or Peruvian shantytown strains). (TIF) [file pone.0015076.s008.tif]

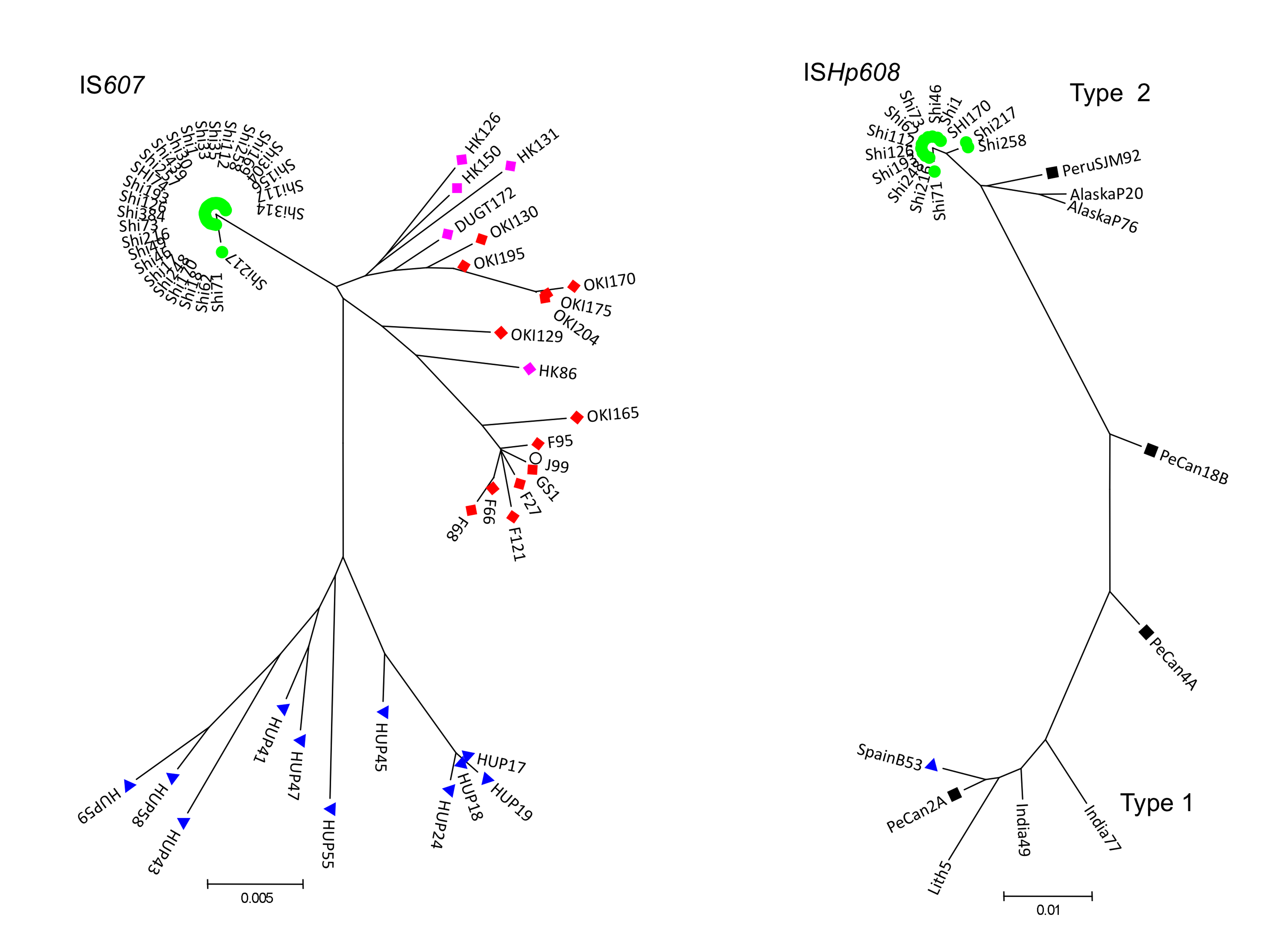

Supplement: Figure S9 — Neighbor joining tree of IS607 and ISHp608 sequences found in Shimaa vs. other strains. The IS607 tree was generated from a central 770 bp segment containing 146 of the orfA transposase gene's 217 codons, 71 of accessory gene orfB's 419 codons. Similarly, the ISHp608 tree was generated from a 654 bp segment containing 88 codons of the 155 codon orfA transposase gene and 101 codons from the 382 codon orfB gene. (TIF) [file pone.0015076.s009.tif]

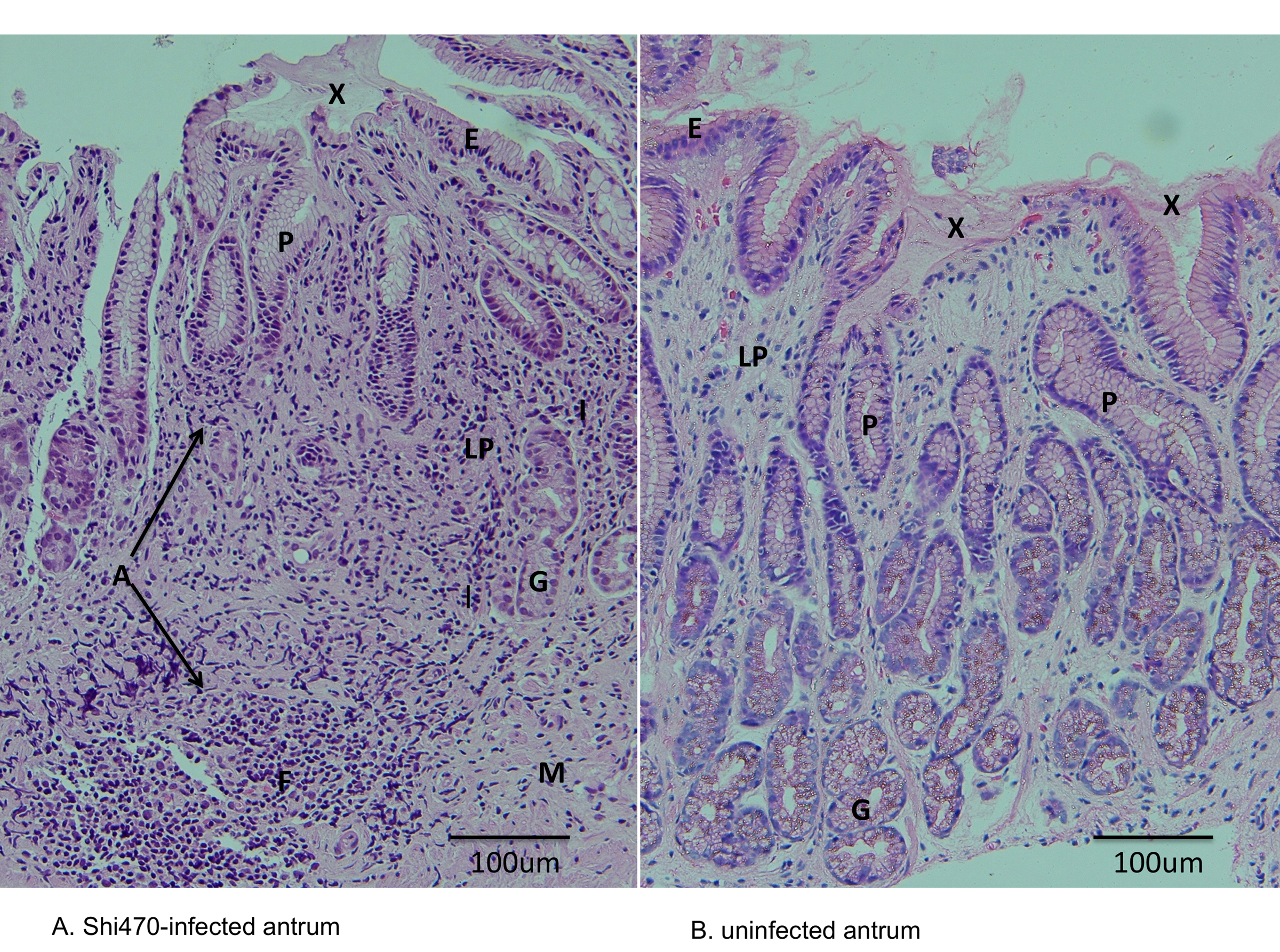

Supplement: Figure S10 — Hematoxylin and eosin stained antrum biopsy sections of Shi470 infected and uninfected Peruvians. A. Gastric biopsy from antrum of Shimaa villager naturally infected with Shi470. Evident here are chronic active antral gastritis with moderate activity (multiple polymorphic neutrophils seen at higher magnification) and moderate chronic inflammation (I) of the lamina propria (LP) extending down to muscularis mucosa (M). Moderate hyperplasia of epithelial cells is seen along the columnar epithelium (E) extending throughout the gastric pits (P). There is moderate glandular atrophy (A) with partial replacement of deep glands with fibrous tissue in areas where the gastric glands (G) should be extending down to the muscularis mucosa. A primary lymphoid follicle is also present as seen by the spherical mass of chronic inflammatory cells (F). Glandular secretions are seen along epithelial surface (X). B. Antrum biopsy section of uninfected antrum from Lima resident. Seen here is uninfected gastric mucosa with columnar epithelial cells (E) and supporting lamina propria (LP) extending down to the start of the muscularis mucosa. The lamina propria of this individual is populated primarily with mesenchymal cells and a few sparse lymphocytes. The stomach antrum contains tightly packed branching tubular glands that open up into irregularly shaped gastric pits (P). The mucus secreting cells of the deep glands play a role in protecting the intestinal mucosa. Note that these glands (G) extend the entirety of the gastric mucosa reaching to the muscularis mucosa at their deepest point. Glandular secretions are seen along the epithelial surface (X). (TIF) [file pone.0015076.s010.tif]

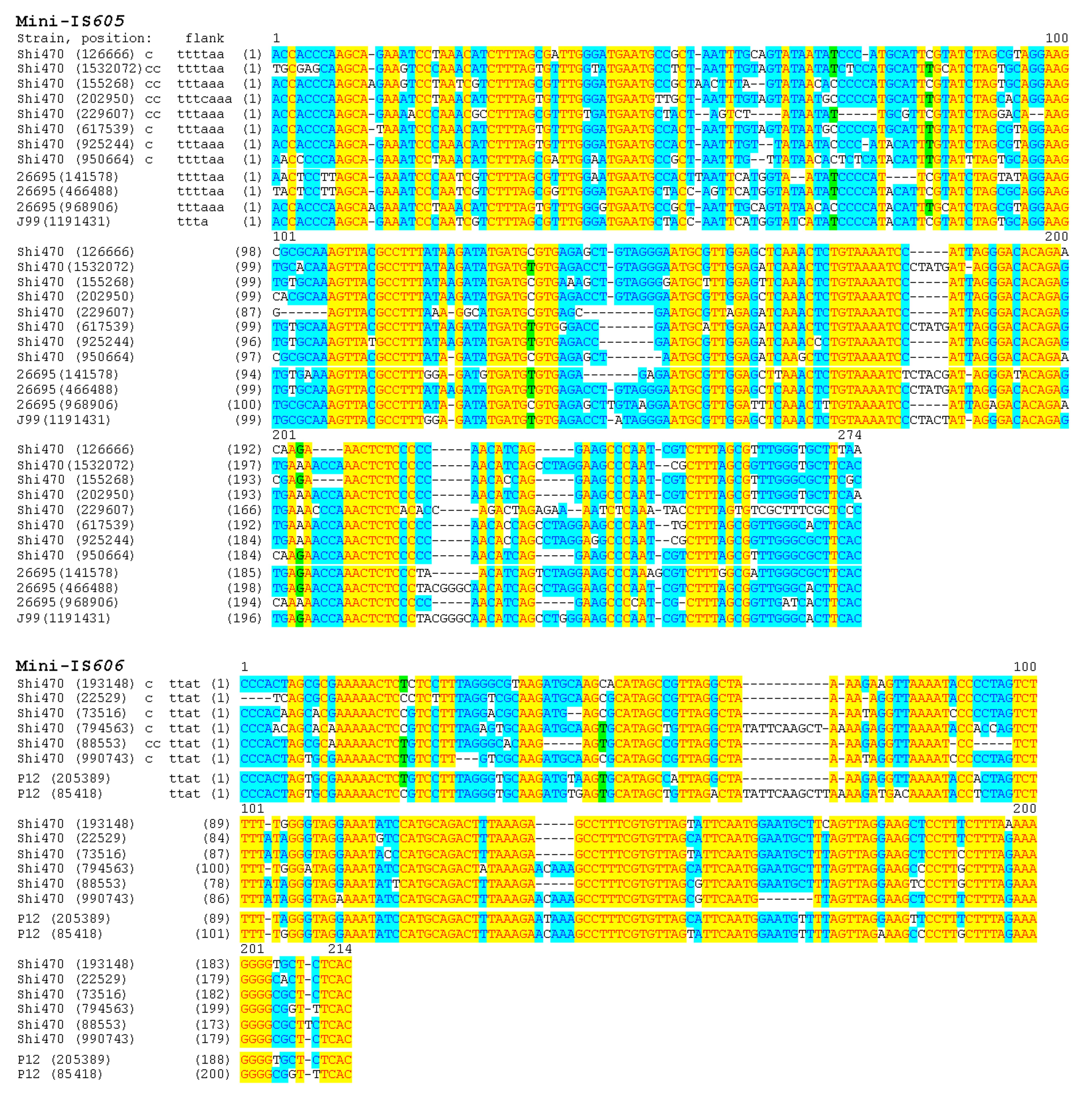

Supplement: Figure S11 — Sequence alignment of mini-IS605 and mini-IS606 elements found in Shi470 genome, relative to those in reference strains. Chromosomal sequences adjacent to mini IS element left ends, positions of left end, and mini-IS orientation [clockwise (c) or counter clockwise (cc)] are indicated. (TIF) [file pone.0015076.s011.tif]
