# Supplementary material for: Helicobacter pylori from Peruvian Amerindians: Traces of Human Migrations in Strains from Remote Amazon, and Genome Sequence of an Amerind Strain
Source: PLoS One. 2010 Nov 29;5(11):e15076. doi: 10.1371/journal.pone.0015076 (PMC2993954; doi:10.1371/journal.pone.0015076)
Supplement: Table S1 — Shi470 genes involved in natural transformation (PDF) [file pone.0015076.s012.pdf]

Table S1. Shi470 genes involved in natural transformation

gene name	orf in Shi470	size (aa)
comB2	*hpsh_00080*	93
comB3	*hpsh_00085*	87
comB4	*hpsh_00090*	787
comB6	*hpsh_00185*	351
comB7	*hpsh_00190*	37
comB8	*hpsh_00195*	247
comB9	*hpsh_00200*	321
comB10	*hpsh_00205*	378
comE	*hpsh_07045*	437
comH	*hpsh_07840*	479
comI	*hpsh_07135*	220
dprH	*hpsh_01730*	266
