# Supplementary material for: Helicobacter pylori from Peruvian Amerindians: Traces of Human Migrations in Strains from Remote Amazon, and Genome Sequence of an Amerind Strain
Source: PLoS One. 2010 Nov 29;5(11):e15076. doi: 10.1371/journal.pone.0015076 (PMC2993954; doi:10.1371/journal.pone.0015076)
Supplement: Table S2 — Outer membrane protein genes in Shi470 (PDF) [file pone.0015076.s013.pdf]

Table S2. Outer membrane protein genes in Shi470

**Major omp family**	**gene name**	**gene identifier in Shi470**
Hop proteins	hopA	*hpsh_01190*
	hopB(alpB)	*hpsh_04810*
	hopC(alpA)	*hpsh_04805*
	hopD	*hpsh_00120*
	hopE	*hpsh*_03325
	hopF	*hpsh_01315*
	hopG	*hpsh_01329*
	hopH (*oipA*)	*hpsh_03300, _03675* (5'-end)[1]
	hopI	*hpsh_05965*
	hopJ/K	*hpsh_02350, _04865*[2]
	hopL	*hpsh_05970*
	hopM/N (*omp5/29*)	*hpsh_01180*[3]
	hopO (*sabB*)	absent
	hopP (*sabA*)	pseudogene *hpsh_03235*
	hopQ	*hpsh_06095*
	hopS (*babA*)	*hpsh_04720*
	hopT (*babB*)	*hpsh_06440*
	hopU (*babC*)	absent
	hopV	*hpsh_07525*
	hopZ	pseudogene *hpsh_00035*
Hor proteins	*horA*	*hpsh_00385*
	horB	*hpsh_00635*
	horC	*hpsh_01680*
	horD	*hpsh_01990*
	horE	*hpsh_02320*
	horF	*hpsh_03505*
	horG	*hpsh_02835*
	horH	*hpsh_05690*
	horI	*hpsh_05720*
	horJ	*hpsh_07525*
	horK	pseudogene *hpsh_07705*
	horL	*hpsh_07930*
**Hof family**	*hofA*	*hpsh_01080*
	hofB	*hpsh_01900*
	hofC	*hpsh_02400*
	hofD	*hpsh_02405*
	hofE	*hpsh_02900*
	hofF	*hpsh_02870*
	hofG	pseudogene *hpsh_04820*
	hofH	*hpsh_06030*
**Hom family**	*homA*	absent
	homB	*hpsh_04925*
	homC	absent

	homD	*hpsh_05550*
**Iron-regulated omp family**		
FecA-like	*fecA1*	*hpsh_03435*
	fecA2	pseudogene *hpsh_02780*
	fecA3	*hpsh_07905*
FrpB-like	*frpB1*	*hpsh_02430*
	frpB2	*hpsh_04830*
	frpB3	pseudogene *hpsh_07760*
**Efflux pump omp family**	*hefA*	*hpsh_03835*
	hefD	*hpsh_05130*
	hefG	*hpsh_06860*
**Other omps**		*hpsh_04395*
		hpsh_03580
		hpsh_03380
		hpsh_03230
		hpsh_05630
		hpsh_07515
		hpsh_01685
		hpsh_02605
		hpsh_05800
		hpsh_07455
		hpsh_08150

Footnotes

1. Different alleles, *hpsh_03675* has frameshift.
2. Identical copies.
3. One copy only.
