# Supplementary material for: Helicobacter pylori from Peruvian Amerindians: Traces of Human Migrations in Strains from Remote Amazon, and Genome Sequence of an Amerind Strain
Source: PLoS One. 2010 Nov 29;5(11):e15076. doi: 10.1371/journal.pone.0015076 (PMC2993954; doi:10.1371/journal.pone.0015076)
Supplement: Table S3 — Primers used for analysis of Shimaa village strains (PDF) [file pone.0015076.s014.pdf]

Table S3. Primers used for analysis of Shimaa village strains

Primer region, name	Primer sequence (5' to 3')
***cagA* (3' region)**	
cagTF	accctagtcggtaatggg
cagTR	gctttagcttctgayacygc (y=c+t)
***cag* PAI Right Junction**	
4584	gttaatacaaaaggtggtttccaaaaatc
5280	ggttgcacgcattttcccttaatc
***vacA* mid region**	
VAmF	ggccccaatgccagtcagtgat
VAmR	gctgttagtgcctaaagaagcat
***vacA* signal sequence**	
VA1-F	atggaaatacaacaaacacac
VA1-R	ctgcttgaatgcgccaaac
***hp0519* region**	
hp518A1F	caagtgtctattataatggaaaaata
hp519/520 F4	attatagcaaaaaatataaaaccat
**IS*605***	
orf19F	ggctgttctagggtcgtgtataac
orf19R	caagctagatgcaatctagctacc
**IS*606***	
FB2-F	ggagggtagttgataagcaaatc
RB3-R	gtttagactttaacaccctacgg
**IS*606*-Shi (remnant)**	
IS606shima-F	gaacaagaaagcaaactgcaac
IS606shima-R	tgttcgcaacgttaatatctctg
**ISHp*607***	
IS607F2	gcatagatatttaagccattaga
IS607R2	tgattatttaaaaatacctaacttacc
**ISHp*608* universal**	
IS608F1univ	ccataacgccttaatagtgtgtc
IS608R	caagctttggagtgatgaagttc
**ISHp*608* type 2**	
608all2F	tatataaaaacaaccacaaca
608all2R	ttttgtcaatcttactctta
**ISHp*609***	
609F1	cacaacaggtattaatgctt
609R1	ctttagctcttgtttccagc
atpA	

atpA1	gcttaaatggtgtgatgtcg
atpA6	cttattcgcccttgcccatt
cysS	
cysS-F	ctacggtgtatgatgacgctca
cysS-R	ccttgtggggtgtccatcaaag
glr	
5733F	cacatcgccgctcgcatga
6564R	aagctttgtgtattctaaaatgcaac
ppa	
ppa8	cccctagaaaatcctattttgataatc
ppa9	agtggtgagctttagcgacgctc
glmM (ureC)	
glmF	tttgggactgatggcgtgaggg
glmR	tcttttaattcttgcattttggattcta
recA	
recA1F	gcgttggtacgccttggggataagcaa
recA4R	gccttgccctagctttttatcctggt
